# A Landscape Analysis of Human SUMOylation

**DOI:** 10.1016/j.mcpro.2026.101571

**Published:** 2026-04-20

**Authors:** Shireen S. Al-Momani, Kerry Ramsbottom, Andrew Collins, Ellen Boswell, Ivo Alexander Hendriks, Michael L. Nielsen, Alfred C.O. Vertegaal, Ronald T. Hay, Zhi Sun, Yasset Perez Riverol, Emily H. Bowler-Barnett, Maria J. Martin, Jun Fan, Ari Sadanandom, Eric W. Deutsch, Juan Antonio Vizcaíno, Andrew R. Jones

**Affiliations:** 1 Institute of Systems, Molecular and Integrative Biology, University of Liverpool, Liverpool, UK; 2 Department of Cellular and Molecular Medicine, Novo Nordisk Foundation Center for Protein Research, Faculty of Health and Medical Sciences, University of Copenhagen, Copenhagen, Denmark; 3 Department of Cell and Chemical Biology, Leiden University Medical Center, Leiden, the Netherlands; 4 Division of Molecular, Cellular and Developmental Biology, School of Life Sciences, University of Dundee, Dundee, UK; 5 Institute for Systems Biology, Seattle, Washington, USA; 6 European Molecular Biology Laboratory, European Bioinformatics Institute (EMBL-EBI), Wellcome Genome Campus, Hinxton, UK; 7 Department of Biosciences, Durham University, Durham, UK

**Keywords:** false localization rate, human SUMO proteome, computational proteomics, mass spectrometry, SUMOylation

## Abstract

SUMOylation is an understudied post-translational modification (PTM) linked to diverse physiological and pathological processes. Advances in enrichment strategies, combined with mass spectrometry (MS), have enabled large-scale mapping of SUMOylation sites. Here, we reanalyzed publicly available MS-based datasets to construct a comprehensive and high-confidence reference set of human SUMOylation sites. Our workflow integrated database searching and scoring through the Trans-Proteomic Pipeline (TPP) with a statistical approach to independently estimate global false localization rate (FLR) of modification sites. SUMOylated lysine sites identified at <5% FLR were classified into three confidence tiers and compared with a high-quality set of non-SUMOylated lysines. The Human SUMO Build comprises 35,721 SUMOylation sites across 6146 proteins. SUMOylated lysines were enriched within intrinsically disordered regions and underrepresented in tightly packed structural elements. These sites exhibited a higher frequency of nearby phosphosites at −2, +1, and +5, and were enriched for disease-linked variants at the modified lysine and the −2 position. Motif analysis revealed canonical, inverted, and novel SUMOylation motifs with flanking amino acid enrichment, including aspartic acid at −2, isoleucine and valine at −1, proline at +1, and glutamic acid at +2. Comparative analysis of SUMOylation and ubiquitination sites revealed that the two modifications frequently target the same lysine residue. Sites exclusively SUMOylated are preferentially located within intrinsically disordered regions, whereas sites exclusively ubiquitinated are enriched in secondary structural elements. Sites modified by both PTMs are enriched more strongly for disease-associated variants at the modified lysine and the −1 position than sites unique to SUMOylation or ubiquitination. Gene Ontology enrichment analysis linked motifs to biological processes, with most motifs contributing to chromatin remodeling, histone modification, and mRNA processing. The Human SUMO Build is publicly available through the PTMeXchange initiative, with data deposited in PRIDE and integrated into UniProtKB and PeptideAtlas to facilitate downstream analyses and predictive modeling.

## Highlights

• We generated a Human SUMO build: 35,721 sites across 6146 proteins.
• SUMOylated lysines are 2.4× more likely to occur in intrinsically disordered regions.
• Exclusively SUMOylated and ubiquitinated sites occupy structurally distinct regions.
• Sites co-modified by SUMO and ubiquitin show the highest disease variants proportion.
• Non-SUMOylated negative set extracted from the same experimental peptide pool.

## Graphical Abstract

**Figure:**
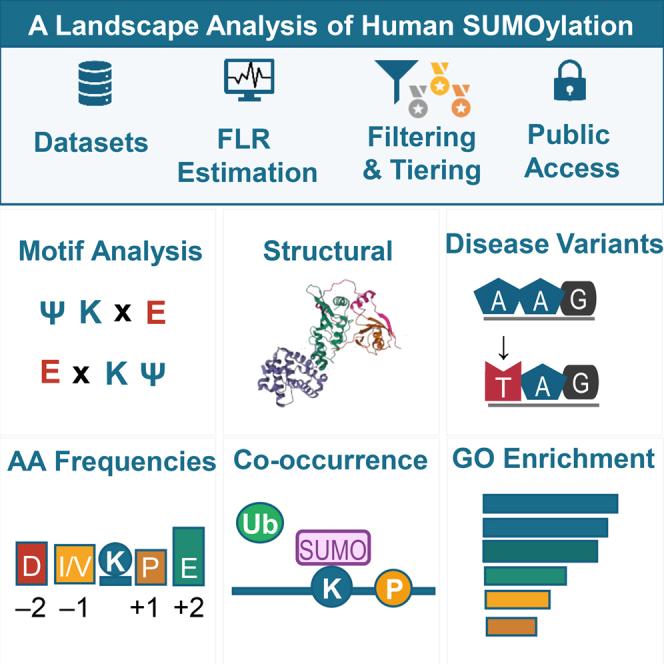

## In Brief

SUMOylation is a post-translational modification implicated in cancers, neurodegenerative disorders, and cardiovascular diseases, yet it remains understudied. By reanalyzing thirteen mass spectrometry datasets using a standardized pipeline with independent false localization rate estimation, we generated a comprehensive SUMOylation build across three confidence tiers. A landscape analysis was performed covering structural properties, motif prediction, phosphorylation co-occurrence, ubiquitin crosstalk, disease variants, and biological pathways. SUMOylated and non-SUMOylated sets are provided as an AI-ready resource, with data openly accessible through major proteomics repositories.

Small Ubiquitin-like MOdifier (SUMO) refers to a family of small proteins that post-translationally attach to protein substrates, altering molecular interactions by adding or masking interaction surfaces, thereby influencing the substrate’s activity, stability, or subcellular localization (1). Protein SUMOylation has attracted significant research focus due to its implications in various diseases, including cancers, neurodegenerative disorders, and cardiovascular diseases, as demonstrated in both human studies and animal models (2, 3, 4, 5, 6, 7, 8, 9, 10). SUMOylation occurs when a cascade involving E1 activating and E2 conjugating enzymes, often with assistance from E3 ligases, catalyzes the covalent isopeptide bond between the C-terminal glycine residue of SUMO and the ε-amine group of a specific lysine residue of the substrate protein. A SUMO-specific protease with isopeptidase activity ensures the reversibility of the reaction by removing SUMO from its target protein. Most SUMO targets are modified in a small percentage at a steady state. The whole pool of a given protein can be SUMOylated quickly, with equilibrium lying to the side of the non-SUMOylated form (1).

In humans, the SUMO protein family is composed of five isoforms, of which SUMO1–3 are the most extensively studied and well-characterized as conjugatable modifiers. SUMO2 is the most abundant isoform and shares approximately 97% sequence identity with SUMO3; the two are therefore often referred to jointly as SUMO2/3. Both SUMO2 and SUMO3 contain internal SUMOylation consensus motifs that mediate the formation of polymeric SUMO chains. In contrast, SUMO1 lacks these internal motifs and primarily functions as a monomer, frequently capping poly-SUMO2/3 chains to regulate the strength of substrate recognition and duration of the SUMOylation signal (11). A recent study has reported that SUMO2/3 conjugation of transcription-associated proteins is rapidly induced under cellular stress conditions, such as oxygen and glucose deprivation, to control cell viability (12). While SUMO1 is primarily conjugated to the nucleocytoplasmic transport regulator RanGAP1 under normal physiological conditions, recent studies have shown that it can also play a role in stress responses, although it may function independent of canonical stress-induced pathways (11, 13).

The presence of SUMO consensus motifs (SCMs), including the classical (ψKxE/D) and inverted (E/DxKψ) motifs, where ψ is a large hydrophobic residue, is considered important for substrate recognition. Variations within the classical motif, including upstream hydrophobic clusters and downstream acidic or phosphorylatable sites, can influence the affinity and specificity of SUMOylation (14). The mere existence of the SCM motif does not solely define a SUMO substrate. Such motifs are also found in many non-SUMOylated proteins (15). Based on past analyses, it has been demonstrated that proteins with at least one [IVL]KxE motif are more than twice as likely to be SUMO targets than proteins without this motif type. Large-scale MS-based SUMO proteomics analysis in humans revealed that more than half of the SUMO substrates are modified at the minimal KxE motif under physiological conditions. However, upon stress, lysine residues were SUMOylated promiscuously with lower adherence to the SUMO consensus motif, resulting in more modified lysines at non-SCM sites (16).

High-throughput tandem MS is commonly used to detect and localize PTM sites on proteins. However, studying SUMO is incompatible with standard MS approaches. There are no trypsin cleavage sites near the SUMO C-termini (trypsin is a protease that cleaves after arginine (R) and lysine (K) unless followed by a proline (P)). Trypsin digestion in the case of SUMO2 and SUMO3 would generate a 32 amino acid sequence attached to the SUMOylated target’s peptide (17). Fragmentation of this 3549.48 Da SUMO tail may dominate the tandem mass spectra and reduce the ability to acquire an identification of the target peptide using search engines, thus preventing efficient analysis by standard MS (18). Two main approaches have been applied to produce proteolytic cleavage remnants short enough for reliable identification. The first approach requires the expression of exogenous SUMO isoforms carrying strategic mutations. Efficient results have been obtained from optimizing the so-called K0 technique, in which all lysines in SUMO2 are mutated to arginine to protect them from Lys-C digestion. Additional arginines are also introduced at positions 88 (Q88R) or 91 (T91R) to leave QQTGG or a diglycine SUMO signature peptide after digestion, respectively (17). The K0 technique has been further optimized by generating stable cell lines, extending to a His10 tag, adding a SUMO purification step before Lys-C digestion, and removing any unconjugated SUMO before purifying His10-SUMO2 modified peptides, allowing for the identification of more than 40,000 mono-SUMO2 acceptor sites (19). Despite being effective in mapping SUMOylation sites, a drawback of using the mutant SUMO approach is that even slight overexpression or the presence of mutated SUMO could increase the risk of generating unnatural SUMO attachments, which may interfere with normal cellular processes and potentially disrupt signal transduction pathways. In addition, this approach requires genetic modification, which is not feasible in either primary cells or patient samples due to technical, biological, and ethical constraints (20, 21). To address this issue, several protocols have been utilized to map endogenous SUMO sites, all of which do not require introducing a mutation or an epitope tag to the SUMO sequence. Hendriks *et al*. reported the largest collection of endogenous SUMO2/3 sites. Their protocol used a peptide-level immunoprecipitation enrichment protocol (8A2 antibody) and further digested purified SUMOylated peptides using Asp-N to leave a DVFQQQTGG signature peptide (22). However, only 14,869 sites were identified, not reaching the number detected in their previous study using the exogenous K0-SUMO method (19).

In this study, we employed a robust computational pipeline to reanalyze publicly accessible human SUMO-enriched MS-based proteomics data. A key component of this pipeline is a statistical approach developed by our team for independently estimating the FLR of modification sites, achieved by searching for modifications on the “decoy” amino acid alanine, which cannot be modified (23). Based on the frequency of observation across independent reanalyses and the FLR threshold applied, each identified SUMOylation site can be assigned to a confidence tier (Gold–Silver–Bronze) (24).

We also constructed a well-curated, high-confidence, and AI-ready reference set of SUMOylated and non-SUMOylated lysine residues. We identified a large “Gold” standard set of SUMOylation sites, alongside a set of non-SUMOylated lysines derived from peptides detected at <1% FDR but lacking any SUMOylation evidence. These two sets were then analyzed and compared in terms of intrinsic disorder, secondary structure, phosphorylation co-occurrence, disease-associated variants, and amino acid preferences in flanking positions. These analyses aim not only to deepen our understanding of the sequence and structural characteristics of SUMOylation but also to provide a high-quality training resource by ensuring that both SUMOylated and non-SUMOylated sets are well defined and characterized—“AI-ready.”

In addition, we conducted a focused analysis to provide insights into the sequence features and biological roles associated with high-confidence SUMOylation events. Overrepresented sequence motifs were extracted to identify common modification patterns. Then, Gene Ontology (GO) enrichment analysis was performed specifically on proteins containing motifs in the most confident tier (Gold set). Finally, to explore potential crosstalk between SUMOylation and ubiquitination, SUMO sites were compared against a large-scale human ubiquitination build, with sites classified into exclusively SUMOylated, exclusively ubiquitinated, and co-modified categories, and further characterized in terms of sequence motifs, intrinsic disorder, secondary structure, disease-associated variants, and proximity to ubiquitination sites. The resulting human “SUMO build” has been made publicly available through the PTMeXchange initiative, with the data deposited in EBI PRIDE and integrated into UniProtKB and PeptideAtlas.

## Methods

### Dataset Collection

Using the search terms ‘SUMO’ and ‘SUMOylation,’ we queried PRIDE/ProteomeXchange (25) and manually curated *Homo sapiens* datasets accessible before June 23, 2024. We selected 13 datasets, where they had documented around 1000 or more SUMO sites in their respective original publications (19, 22, 26, 27, 28, 29, 30, 31, 32, 33, 34, 35). Metadata were extracted, including information on identified SUMO isoforms, mutation types, SUMO footprints (mass remnants) searched, experimental conditions, cell lines, and a numerical overview of SUMOylated sites and proteins from the original analyses, as shown in Table 1.

**Table 1:** Summary of the 13 published SUMO proteomics datasets reanalyzed in this study

Dataset identifier	SUMO isoform	Mutation	Footprint	Experimental condition	Cell line	Original study (Site/Protein count)	Ref
PXD001061	SUMO2	SUMO2-K0-Q87R	QQTGG and pQQTGG	MG-132, PR-619 and heat shock (43 °C)	HeLa	4361/1606	(28)
PXD001281	SUMO2	SUMO2-T90K	diGly	Heat shock (43 °C)	HEK293	1007/539	(35)
PXD004927	SUMO2	SUMO2-K0-8KR-Q87R	QQTGG and pQQTGG	Proteotoxic stress (MG-132 & bortezomib) and heat shock (43 °C)	HeLa and U2OS	40,765/6747	(19)
PXD005296	SUMO2	SUMO2-K0-8KR-Q87R	QQTGG and pQQTGG	CDKi treatment	HeLa and U2OS	8044/2104	(19)
PXD006361	SUMO2	SUMO2-K0-Q87R	QQTGG and pQQTGG	Replication stress (mitomycin C (MMC) and hydroxyurea (HU))	U2OS	1590/576	(34)
PXD006545	SUMO3	SUMO3-Q87 R/Q88N	NQTGG	MG-132 and heat shock (42 °C)	HEK293	>8000/3229	(31)
PXD007629	SUMO2	SUMO2-T90K	diGly	Hinokiflavone treatment	HEK293	924/543	(33)
PXD008003	SUMO2/3^a^	NA	DVFQQQTGG	Proteasome inhibition (MG-132 and heat shock (43 °C))	HEK293, HeLa, and U2OS	14,869/3981	(22)
PXD011932	SUMO3	SUMO3-Q87 R/Q88N	NQTGG	E3 SUMO ligase PIAS1 overexpression	HEK293	983/544	(30)
PXD028050	SUMO2 and SUMO1^b^	SUMO1-T95 K and SUMO2-T90K	diGly	Pluripotent state	ChiPSC4	976/427	(32)
PXD022367	SUMO2/3^a^	NA	DVFQQQTGG	Standard cell culture conditions	U2OS	1606/700	(27)
PXD023841	SUMO3	SUMO3-Q87 R/Q88N	NQTGG	Cell cycle and cell proliferation with PIAS knockout	HEK293	1422/530	(29)
PXD034631	SUMO1 and SUMO2^b^	SUMO1-T95 K and SUMO2-T90K	diGly	Epstein-Barr virus infection	AGS	1726/828	(26)

a The endogenous approach used for SUMO site identification cannot distinguish between SUMO2 and SUMO3 due to their high sequence similarity.

b Cell lines were genetically engineered to stably express SUMO1 and SUMO2 mutants.

### Peptide and Protein Identification and Modification Localization

#### Removing SUMO Fragment Peaks from Raw Data

The datasets were first processed by converting raw files to the open standard mzML format, using the ThermoRawFileParser tool (36). One challenge with confident SUMO identification is that our preferred search engine (Comet) does not have the ability to score ions resulting from fragmentation of the SUMO side chain. As these peaks can be quite abundant in spectra, particularly for larger SUMO footprints, they can cause lower confidence in peptide-spectrum matches (PSMs) or SUMO site localization. To calculate the percentage of MS/MS spectra featuring SUMO remnant fragment ion peaks, the Pyteomics Python library (37) was used to parse the mzML files and count MS/MS spectra exhibiting those specific peaks. Each spectrum was evaluated by comparing the relative intensity of the fragment ion peak to the base peak, considering only those with a relative intensity greater than 0.1.

To enhance the identification of SUMOylated peptides, mzML files in datasets with notably high percentages of MS/MS spectra containing SUMO remnant fragment ion peaks underwent a filtering procedure before performing Comet searches. The Fragment Ion Calculator (http://db.systemsbiology.net/proteomicsToolkit/FragIonServlet.html) was used to acquire monoisotopic masses in Daltons for fragment ions generated from the fragmentation of SUMO-2 remnants (QQTGG and pyro-QQTGG), SUMO-3 remnant (NQTGG), and SUMO2/3 remnant (DVFQQQTGG) for datasets PXD004927, (PXD006545 and PXD023841), and (PXD008003 and PXD022367), respectively. The mass error was calculated for each fragment ion by multiplying its monoisotopic mass by the precursor mass tolerance. We then adjusted the monoisotopic mass by adding or subtracting this mass error. Finally, to refine the MS/MS spectra, we employed the mzR package (38) to identify and remove peaks with mass-to-charge (m/z) ratio values that matched the adjusted monoisotopic mass of the fragment ion and produce a filtered mzML file.

#### Preparing Protein and Peptide Search Databases

For most datasets, a protein database was used with *in silico* digestion performed directly by Comet during database searching. This approach was applied to the following datasets: PXD001061, PXD001281, PXD004927, PXD005296, PXD006361, PXD006545, PXD007629, PXD011932, PXD023841, PXD028050 (Lys-C processed samples), and PXD034631 (Lys-C processed samples). To prepare the human protein database, the human proteome (one protein sequence per gene) was downloaded from UniProt Release 2022_03. Contaminants commonly found in proteomics experiments (https://www.thegpm.org/crap/) were added to the human proteome, then decoy sequences were generated using the de Bruijn decoy generator (39). For the PXD034631 dataset, a second protein database was created by including 84 Epstein–Barr virus (EBV) protein sequences, sourced from the PXD034631 ProteomeXchange submission, to the human proteome and contaminants before generating the decoy sequences.

For datasets requiring up to eight missed cleavages, which exceeds Comet’s *in silico* digestion limit of five missed cleavages, peptide databases were generated. This approach was used for PXD022367, PXD008003, PXD028050 (Lys-C/Glu-C processed samples), and PXD034631 (Lys-C/Glu-C processed samples). To generate peptide databases, the human protein database underwent *in silico* Lys-C digestion using custom Python code. This involves cleaving after lysine (K) residues, unless followed by a proline (P), with allowance for up to eight missed cleavages. The removal of the leader methionine residue resulting from proteolytic processing was also considered. If a protein sequence lacked a Lys-C site, the entire sequence was appended to the list of Lys-C digested peptides. For datasets PXD008003 and PXD022367, Lys-C digested peptides were further cleaved before aspartic acid (D) and glutamic acid (E) to simulate the digestion with Asp-N + N-terminal Glu proteases. For datasets PXD028050 and PXD034631, Lys-C digested peptides were cleaved after D/E to simulate digestion using Glu-C proteases. In both cases, up to eight missed cleavages were allowed.

#### The Trans-Proteomic Pipeline (TPP)

Software tools integrated into the TPP pipeline version 6.3.2 (40) were then used to reanalyze mzML files. First, the Comet database search engine Release 2023.01 rev. 2 (41) was used to search mass spectra against the human databases. Carbamidomethylation of cysteine: 57.021464 was set as a fixed peptide modification for all datasets. To enhance statistical power, phosphorylation on serine, threonine, and tyrosine (STY) was not included as a variable modification in all datasets, even though in many original searches this had been included, as SUMO can commonly occur proximally to phosphorylation sites. Our pilot results suggested that more SUMO identifications were made by excluding this from searches. Other search parameters were selected to match as closely as possible the parameters from the original analyses. A summary of search parameters used in Comet database searching is shown in Table 2. To validate peptide assignments to spectra, PeptideProphet (42) was used to model the score and assign the probability of being correct for each PSM. Probabilities and mixture models were then further refined using the iProphet (InterProphet) tool (43). Finally, PTMProphet (44) was used to apply a statistical model to predict which modification sites are most probably localized.

**Table 2:** Comet search parameters used for the reanalyses of selected datasets

Dataset identifier	Peptide mass error tolerance	Fragment mass error tolerance	Enzyme	Missed cleavages	Variable modification: Mass in Daltons
PXD001061	6 ppm	0.02 Da	Trypsin	Three	Oxidation of methionine: 15.9949, protein N-terminal acetylation: 42.0106, peptide N-terminal carbamylation: 43.00581, SUMOylation (QQTGG): 471.20776^ab^, SUMOylation (Pyro-QQTGG): 454.18121^a^^b^^c^
PXD001281	6 ppm	0.5 Da	Lys-C or Lys-C/Glu-C	Three (Lys-C) or Five (Lys-C/Glu-C)	Oxidation of methionine: 15.9949, protein N-terminal acetylation: 42.0106, SUMOylation (diGly): 114.043^a^^b^
PXD004927	5 ppm	0.02 Da	Trypsin	Four	Oxidation of methionine: 15.9949, protein N-terminal acetylation: 42.0106, SUMOylation (QQTGG): 471.20776^a^^b^, SUMOylation (Pyro-QQTGG): 454.18121^a^^b^^c^
PXD005296	5 ppm	0.02 Da	Trypsin	Four	Oxidation of methionine: 15.9949, protein N-terminal acetylation: 42.0106, SUMOylation (QQTGG): 471.20776^a^^b^, SUMOylation (Pyro-QQTGG): 454.18121^a^^b^^c^
PXD006361	6 ppm	0.02 Da	Trypsin	Two	Oxidation of methionine: 15.9949, protein N-terminal acetylation: 42.0106, N-pyro-glutamine: −17.026549, SUMOylation (QQTGG): 471.20776^a^^b^, SUMOylation (Pyro-QQTGG): 454.18121^a^^b^^c^
PXD006545	5 ppm	0.02 Da	Trypsin	Three	Oxidation of methionine: 15.9949, protein N-terminal acetylation: 42.0106, asparagine and glutamine deamidation: 0.98402, ubiquitination of lysine (GG): 114.043, SUMOylation (NQTGG): 457.192118^a^^b^
PXD007629	6 ppm	0.5 Da	Lys-C or Lys-C/Glu-C	Three (Lys-C only) Five (Lys-C/Glu-C)	Oxidation of methionine: 15.9949, protein N-terminal acetylation: 42.0106, SUMOylation (diGly): 114.043^a^^b^
PXD008003	5 ppm	0.02 Da	Lys-C/Asp-N	Eight^d^	Oxidation of methionine: 15.9949, protein N-terminal acetylation: 42.0106, SUMOylation (DVFQQQTGG): 960.4301^a^
PXD011932	10 ppm	0.01 Da	Trypsin	Two	Oxidation of methionine: 15.9949, protein N-terminal acetylation: 42.0106, asparagine and glutamine deamidation: 0.98402, and SUMOylation (NQTGG): 457.192118^a^^b^
PXD028050	6 ppm	0.5 Da	Lys-C or Lys-C/Glu-C	Three (Lys-C) or Eight (Lys-C/Glu-C)^d^	Oxidation of methionine: 15.9949, protein N-terminal acetylation: 42.0106, SUMOylation (diGly): 114.043^a^^b^
PXD022367	5 ppm	0.02 Da	Lys-C/Asp-N	Eight^d^	Oxidation of methionine: 15.9949, protein N-terminal acetylation: 42.0106, peptide N-terminal pyroglutamate from Q: −17.026549, SUMOylation (DVFQQQTGG): 960.4301^a^
PXD023841	10 ppm	0.02 Da	Trypsin	Three	Oxidation of methionine: 15.9949, protein N-terminal acetylation: 42.0106, SUMOylation (NQTGG): 457.192118^a^^b^
PXD034631	10 ppm	0.02 Da	Lys-C or Lys-C/Glu-C	Three (Lys-C only) or Eight (Lys-C/Glu-C)^d^	Oxidation of methionine: 15.9949, protein N-terminal acetylation: 42.0106, SUMOylation (diGly): 114.043^a^^b^

a Modification was also searched on alanine in addition to lysine to allow for the independent estimation of global FLR using the decoy FLR method.

b Not allowed to occur at the C-terminus of peptides.

c The N-terminal QQTGG glutamine can cyclize to pyroglutamate under alkaline conditions.

d A peptide database was used, and the “allowed_missed_cleavage” parameter was set to 0 in the comet.params file.

### Independent Estimation of Global False Localization Rate (FLR)

TPP search results in mzIdentML format were then analyzed using the mzidFLR pipeline v2.0.0 (https://github.com/PGB-LIV/mzidFLR). The mzidFLR workflow involves the following steps: First, high-confidence PSMs are identified by calculating the false discovery rate (FDR) at the PSM level and filtering PSMs based on an FDR cutoff of 0.01 (1%). The workflow then expands PSM information into a site-based format and filters the data for the specified modification mass. For SUMOylation, the specified modification mass is the SUMO footprint mass rounded to two decimal places. To estimate the global FLR, the decoy amino acid FLR method was applied. An additional step of binomial adjustment is applied to produce more accurate estimates of the FLR, adjusted for evidence coming from multiple redundant observations of the same sites in different mass spectra. Finally, data is collapsed by “peptidoform-site,” i.e., one row of the file is all PSMs supporting one given SUMO site within a peptidoform (peptide sequence and all modification positions), then all possible PTM positions are mapped onto corresponding proteins (23, 24).

### Post-mzidFLR Data Processing

To ensure standardized and comparable outputs across datasets, a custom Python script was written to process and refine SUMOylation site identifications.

#### Site-Based Merging and Filtering

The peptidoform-site mzidFLR output was collapsed per site, such that each row represents a unique SUMOylation site on a specific protein. Sites with an FLR below 5% were retained to reduce potential false positives, with removal of contaminants and exclusion of decoy SUMOylation sites mapped to alanine residues. For datasets containing multiple SUMO footprints or alternative digestion strategies, the script merges results from parallel searches before site-level collapsing.

#### Filtering SUMO Sites Derived From Asp-N

In the endogenous datasets (PXD008003 and PXD022367), SUMO sites derived from Asp-N were filtered to permit SUMOylation on peptide C-terminal lysine residues only when the preceding residue was aspartic acid (D) or glutamic acid (E). This is because Asp-N cleaves N-terminal to D or E residues, so any lysine at the C-terminus is part of the actual peptide sequence, not a result of the cleavage rule (as is the case with trypsin), and may genuinely be SUMOylated.

#### The Gold–Silver–Bronze Tiering System

Identified SUMO sites were combined and classified into three sets, with different levels of quality: Gold, Silver, and Bronze, based on the frequency of observation across independent reanalyses and the FLR threshold applied. The Gold set includes sites identified in two or more datasets with an FLR below 1%. The Silver set includes sites found in one dataset with an FLR below 1%. The Bronze set includes sites that do not meet the criteria for inclusion in the Gold or Silver sets but still exhibit an FLR below 5% (24).

### Structural Properties of SUMOylation Sites

#### Intrinsic Disorder Analysis

Per-residue disorder scores were predicted using Metapredict version 3 (45). A threshold of 0.5 was applied, classifying residues with scores >0.5 as disordered and those with scores ≤0.5 as ordered, following Metapredict v3 guidelines.

To quantify the relative preference of SUMOylated lysine residues for intrinsically disordered regions (IDRs) compared to ordered regions, we calculated an odds ratio based on the observed and expected proportions of lysines. The odds ratio was calculated using the following equation:

$$Odds\;Ratio=\frac{p_{Disordered}^{obs}/p_{Disordered}^{\exp}}{p_{Ordered}^{obs}/p_{Ordered}^{\exp}}$$

Where $p_{Disordered}^{obs}$ and $p_{Ordered}^{obs}$ represent the observed proportions of SUMOylated lysines in disordered and ordered regions, respectively, and $p_{Disordered}^{\exp}$ and $p_{Ordered}^{\exp}$ represent the corresponding expected background proportions in the human proteome.

An odds ratio greater than 1 indicates a preference for SUMOylated lysines to occur in disordered regions, a value of 1 indicates no preference, and a value less than 1 indicates a preference for ordered regions.

#### Solvent Accessibility Analysis

Relative Solvent Accessibility (rASA) values were computed from AlphaFold-derived structures (46) using the Accessibility module in OpenStructure version 2.7 with the NACCESS backend (47). Lysine residues with at least 20% rASA were classified as exposed (48).

#### Secondary Structure Analysis

Annotated secondary structural elements (β-strands, helices, turns, and coiled coils) of reviewed proteins were retrieved from UniProt Release 2025_02. Lysine residues were mapped to their corresponding UniProt identifiers along with their associated secondary structural annotations. The proportion of lysines falling within each structural element was then calculated for each set.

### Disease-Associated Variant Analysis

Human amino acid variants were obtained from UniProtKB Release 2025_02, using the UniProt Proteins API to download all variants, including those with associated diseases. For each lysine site, all variants mapping to the lysine residue and its ±2 flanking positions were retrieved. Synonymous substitutions were excluded to focus on amino acid-altering mutations. The proportion of disease-associated variants at each relative position was then calculated by dividing the number of disease-associated variants by the total number of variants observed at that position.

### SUMOylation and Phosphorylation Co-Occurrence Analysis

Two analyses were performed to investigate the co-occurrence of SUMOylation and phosphorylation in protein sequences. Both analyses were performed using a high-confidence phosphorylation dataset in which each phosphosite is supported by at least five independent pieces of evidence from both PhosphoSitePlus and PeptideAtlas to ensure a lower false discovery rate compared to other reported phosphosites (49).

#### Phosphosite Proximity Profiling

The analysis was conducted to determine whether SUMOylated lysines in the Gold set are more likely to have nearby phosphosites compared to non-SUMOylated lysines. For each lysine residue, the number of phosphosites located within a ±10 amino acid window on the same protein was counted. Lysines were then grouped based on the number of nearby phosphosites. For each group, the relative frequency was calculated by dividing the number of lysines in that group by the total number of lysines analyzed.

#### Phosphosite Positional Enrichment

The relative positions of phosphosites around lysine residues were evaluated to determine whether phosphosites preferentially occur at specific positions relative to SUMOylated lysines in the Gold set compared to the non-SUMOylated set. For each lysine, phosphosites located within a ±10 residue window on the same protein were identified, and their relative positions were calculated. To obtain the percentage frequency at each relative position, the number of phosphosites observed at that position was divided by the total number of phosphosites detected within the ±10 residue window for each set.

### Analysis of Amino Acids Adjacent to SUMOylated Sites

Amino acids were identified at positions −2, −1, +1, and +2 relative to lysine residues. The frequency of each amino acid at each proximal position was normalized by dividing it by the total number of observations. The resulting values were then multiplied by 1000 to ensure that the total sum of amino acid counts at each proximal position equaled 1000. Next, the normalized amino acid counts were divided by the normalized counts in the human proteome to fix the proteome's counts at 1.

### SUMOylation Motif Prediction

PyMotif-x (https://github.com/dengzq1234/PyMotif-x), the Python implementation of Motif-x (50), was used to extract overrepresented sequence motifs. The process for creating the foreground and background (reference) 15-mer sequences was as follows: for the foreground 15-mer sequences, pre-aligned 15-mer peptides were centered on each lysine known to be SUMOylated and extended seven residues towards the N-terminus and seven residues towards the C-terminus. If the site was located less than seven residues from the N/C-terminus, the 15-mer was completed with the letter “X” to reach a length of 15 residues. The 15-mer peptides were then filtered for redundancy to only include unique sequences. For the background dataset, 15-mer peptides were centered on every lysine residue in the human proteome and formatted according to the same procedure described in the previous section, but without the filtering for redundancy step. The occurrence threshold (i.e., the minimum number of times a particular motif must appear to be considered overrepresented) was set to ≥ 25, and the significance threshold was set to ≤ 0.000001 to maintain a low false positive rate.

### Disease-Associated Variant Enrichment within SUMOylation Motifs

Lysine residues in the SUMOylated Gold set and the non-SUMOylated set were grouped by motif. Variants mapping to each lysine residue were retrieved using the UniProtKB Release 2025_02 dataset described in the Disease-Associated Variant Analysis section. For each motif, the enrichment ratio was computed as the proportion of disease-associated variants in the SUMOylated set divided by the corresponding proportion in the non-SUMOylated set.

### Gene Ontology Enrichment Analysis

A custom Python script was written to extract proteins associated with each motif in the Gold set. Next, we utilized the ClusterProfiler R package (v. 4.6.2) (51) to conduct GO enrichment analysis. The background set (universe set) was defined as the group of genes encoding all the SUMOylated proteins identified in the Gold–Silver–Bronze set. Using the pheatmap R package version 1.0.12 (https://cran.r-project.org/web/packages/pheatmap/), we generated a heatmap to visualize the relationship between motifs and their corresponding GO terms. The heatmap was optimized for improved data interpretation by incorporating clustering and tree-cutting techniques. Only terms significantly enriched (p-adjust value < 0.05) for at least one motif were included. Redundant terms with a similarity score greater than 0.3 were grouped, and a representative term was selected using the simplify() function from the ClusterProfiler package.

### SUMOylation and Ubiquitination Comparative Analysis

Ubiquitination sites were obtained from the human ubiquitin build generated as part of the PTMeXchange initiative (52). Lysine residues from both the ubiquitin and the SUMO builds were combined and classified into three categories: sites exclusively SUMOylated (SUMO-unique), sites exclusively ubiquitinated (Ub-unique), and sites modified by both SUMO and ubiquitin (SUMO–Ub overlap).

#### Motif Prediction

The PyMotif-x workflow and parameter settings described in the “SUMOylation Motif Prediction” section were applied to three additional foreground 15-mer sequences constructed from sites in SUMO-unique, Ub-unique, and SUMO–Ub overlap categories. Only motifs appearing in at least 0.5% of the total sites within each set (corresponding to ≥73, ≥105, and ≥467 foreground matches in the SUMO-unique, SUMO–Ub overlap, and Ub-unique sets, respectively) were retained for comparison.

#### Intrinsic Disorder

The Metapredict-based disorder classification described in the “Intrinsic disorder analysis” section was applied to sites in the SUMO-unique, Ub-unique, and SUMO–Ub overlap sets.

#### Proximity and Positional Enrichment Analysis

The co-occurrence of SUMOylation and ubiquitination was investigated using the same workflow described for the SUMOylation–phosphorylation analysis. Only sites classified as Gold in the human SUMO and ubiquitin builds were included. The ubiquitin Gold set comprised 44,191 high-confidence ubiquitination sites across 8092 proteins. Because ubiquitin targets lysine residues, the central lysine (position 0) was included in the positional enrichment analysis, resulting in 21 relative positions (−10 to +10).

#### Secondary Structure

The proportion of sites within each structural element for the SUMO-unique, ubiquitin-unique, and SUMO–Ub overlap sets was determined as detailed in the “Secondary structure analysis” section.

#### Disease-Associated Variant

The variant-mapping and proportion calculation described in the “Disease-Associated Variant Analysis” section was applied to each lysine residue in the SUMO-unique, SUMO–Ub overlap, and Ub-unique sets, and to their ±2 flanking positions.

### Experimental Design and Statistical Rationale

Appropriate statistical tests were applied with correction for multiple comparisons to ensure reliable and reproducible findings. In the intrinsic disorder analysis, we assessed whether the differences in disorder proportions between SUMOylated and non-SUMOylated lysines were statistically significant. Chi-squared tests were performed as pairwise comparisons between each SUMOylated set and the non-SUMOylated set. Bonferroni correction was applied to adjust for multiple comparisons. In the solvent accessibility analysis, the distributions of rASA values between SUMOylated and non-SUMOylated lysines were compared using a two-sided Mann–Whitney U test. In the secondary structure analysis, we assessed whether the differences in the overall secondary structure proportions between SUMOylated and non-SUMOylated lysines were statistically significant. Chi-squared tests were performed as pairwise comparisons between each SUMOylated set and the non-SUMOylated set. Bonferroni correction was applied to adjust for multiple comparisons.

In the disease-associated variant analysis, the proportions of disease-associated variants at flanking positions (−2 to +2) were compared between each SUMOylated set (Gold, Gold–Silver, and Gold–Silver–Bronze) and the non-SUMOylated set using the chi-squared test of independence. A Benjamini–Hochberg procedure was applied to control the false discovery rate across 15 pairwise comparisons (3 sets × 5 positions). In the SUMOylation and phosphorylation co-occurrence analysis, a chi-squared test was performed at each relative position to compare the number of phosphosites between the Gold and non-SUMOylated sets. To account for multiple comparisons across the 20 relative positions analyzed (−10 to −1 and + 1 to +10), *p*-values were adjusted using the Benjamini–Hochberg procedure to control the false discovery rate.

In the analysis of amino acids adjacent to SUMOylated sites, proximal amino acid frequencies in the Gold set were compared to the non-SUMOylated set using the chi-squared test. A significance threshold of ≤0.0006 (0.05/84) was applied using the Bonferroni correction for multiple comparisons. The total number of comparisons was calculated as 4 proximal positions × 20 amino acids, plus one additional possibility for the absence of an amino acid, resulting in 4 × 21 = 84. Proximal amino acid frequencies in each protease protocol were compared to their non-SUMOylated counterparts using the chi-squared test. A significance threshold of ≤0.00012 (0.05/252) was applied using the Bonferroni correction for multiple comparisons. The total number of comparisons was calculated as 4 proximal positions × 20 amino acids, plus one additional possibility for the absence of an amino acid × 3 (SUMOylated vs not SUMOylated for each protease protocol), resulting in 4 × 21 x 3 = 252. In the analysis of disease-associated variant enrichment within SUMOylation motifs, Fisher’s exact test was performed to assess whether these proportions differed between the SUMOylated and non-SUMOylated sets. *p*-values were adjusted using the Benjamini–Hochberg procedure to control the false discovery rate.

For the Comparative Analysis of SUMOylation and Ubiquitination Sites, statistical testing was performed separately within each sub-analysis. In the Intrinsic disorder section, the proportion of sites located within intrinsically disordered regions for each set was compared with the proteome background (32.2%) using chi-squared tests with Benjamini–Hochberg correction for multiple testing. In the Secondary structure section, each lysine site category was compared to the proteome baseline for each structural element using chi-squared tests with Benjamini–Hochberg correction. In the Disease-associated variant section, the proportions of disease-associated variants at each of the five relative positions (−2 to +2) were compared across site categories using pairwise 2 × 2 chi-squared tests. *p*-values were adjusted using the Benjamini–Hochberg procedure across all 15 tests (five positions × three pairwise category comparisons). In the Proximity and positional enrichment section, chi-squared tests were performed at each relative position to compare the number of ubiquitination sites between the Gold and non-SUMOylated sets. To account for multiple comparisons across the 21 relative positions analyzed (−10 to +10), *p*-values were adjusted using the Benjamini–Hochberg procedure to control the false discovery rate.

## Results

### Creation of a Human SUMO Atlas Through Reanalysis

The human SUMO build (Supplemental Table S1) comprises 35,721 SUMOylated sites from 6146 SUMOylated proteins, generated by combining results from 13 datasets and selecting only sites with an FLR below 5% in at least one dataset. A summary of SUMOylation sites and SUMOylated proteins identified at 5% and 1% FLR thresholds for each dataset is provided in Table 3.

**Table 3:** Count of SUMO sites and SUMOylated proteins identified below 1% and 5% FLR for each dataset

Dataset identifier	5% FLR Site count^a^	5% FLR Protein count	5% FLR decoy PTM^b^	1% FLR Site count^a^	1% FLR Protein count	1% FLR decoy PTM^b^
PXD001061	4187	1567	162	2592	1164	20
PXD001281	1042	595	41	606	394	4
PXD004927	27,964	5229	1309	18,221	4361	165
PXD005296	6391	1803	312	4479	1474	38
PXD006361	1827	678	71	995	442	6
PXD006545	8586	2857	283	5297	2170	34
PXD007629	615	396	28	455	310	4
PXD008003	11,110^c^	3390	548	5255^c^	2161	47
PXD011932	957	564	35	454	310	3
PXD022367	1337^c^	664	93	728^c^	424	6
PXD023841	2908	1311	107	1962	1064	15
PXD028050	642	317	35	200	127	0
PXD034631	1743	884	86	1114	647	10

a After removing SUMOylated Ala hits.

b SUMOylated Ala hits: Identifications of the SUMO footprint on the decoy amino acid alanine, known as a ‘Decoy PTM’. Such hits are considered false localizations and should be removed from downstream analyses, as alanine is not a true SUMOylation site.

c After excluding Asp-N-derived SUMO sites on peptide C-terminal lysines, where the following residue was not aspartic acid or glutamic acid, in PXD008003 (152 and 35 sites) and in PXD022367 (7 and 2 sites) at 5% and 1% FLR thresholds, respectively.

To reduce noise and enhance the identification of peptides and proteins, we removed SUMO remnant peaks for mzML files from datasets with notably high percentages (>30%) of MS^2^ spectra featuring SUMO remnant fragment ion peaks: PXD023841 (71%), PXD004927 (65%), PXD022367 (54%), PXD006545 (48%), and PXD008003 (33%). Counts of SUMOylation sites and SUMOylated proteins in these datasets prior to filtering are provided in Supplemental Table S2. This process led to modest improvements in SUMO site and protein identification across all filtered datasets.

The endogenous (DVFQQQTGG) datasets showed the most significant percentage increases in SUMO sites at both 5% and 1% FLR thresholds: PXD008003 (+13.4% and +19.0%, respectively) and PXD022367 (+12.8% and +17.8%, respectively). Datasets using the NQTGG SUMO tag demonstrated moderate improvements: PXD023841 (+8.3% and +5.6%, respectively) and PXD006545 (+4.6% and +5.1%, respectively), while the K0 dataset PXD004927 (QQTGG) showed minimal increases (+1.14% and +0.61%, respectively).

**Fig. 1:**
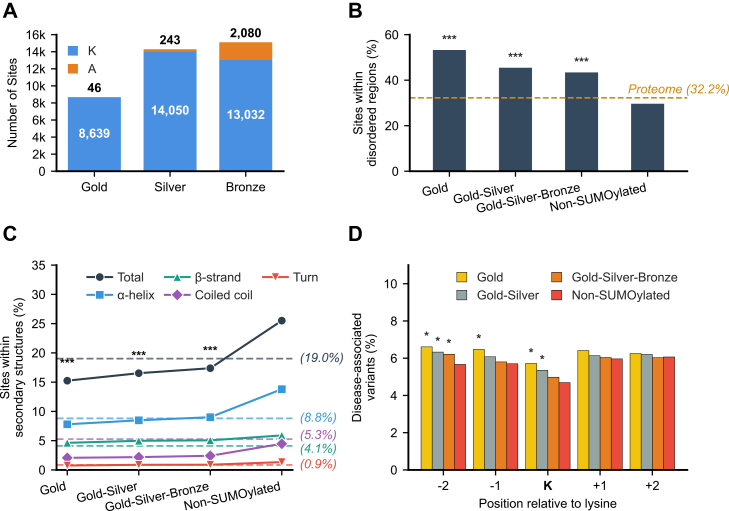
**Site counts and associated features with progressive inclusion of lower-confidence sites**. *A*, count of sites detected with SUMOylation on lysine and on the decoy amino acid alanine in the three quality sets (Gold, Silver, Bronze). *B*, proportions of lysine residues with a per-residue disorder score >0.5 that are SUMOylated in the Gold, Gold–Silver, and Gold–Silver–Bronze sets and not SUMOylated in the non-SUMOylated set. The *dashed yellow baseline* represents the expected proportion of disorder in the human reference proteome. *C*, proportions of SUMOylated and non-SUMOylated lysines located within each secondary structural element (α-helix, β-strand, coiled coil, and turn). The *solid black line* indicates the total proportion in any secondary structure. Dashed baselines represent expected proportions in the human reference proteome, with the *black dashed line* showing the overall proportion of structured regions, and each color-coded *dashed line* matching the color of its corresponding structural element’s *solid line*. *D*, proportions of disease-associated variants at the lysine site and at flanking positions −2, −1, +1, and +2 in SUMOylated and non-SUMOylated sets. Asterisks denote statistical significance (∗*p*< 0.05, ∗∗∗*p*< 0.0001).

Sites in the SUMO build were classified into three quality sets (Gold, Silver, and Bronze) based on the number of times they were observed across independent reanalyses and the FLR cutoff applied. The Gold set contains 8639 high-confidence sites considered to be of high quality on 2742 proteins. The sites included are identified in two or more datasets with an FLR of less than 1%. The Silver set comprises 14,050 sites on 4461 proteins. The sites included are present in one dataset with an FLR of less than 1%. The Bronze set comprises 13,032 sites on 4662 proteins. The sites included do not meet the criteria for inclusion in the Gold or Silver sets but still exhibit an FLR of less than 5% in one or more datasets. Results from the Gold–Silver–Bronze classification are shown in Figure 1*A*.

To highlight the progression from the most stringent (Gold) to the least stringent (Bronze) levels of confidence, the quality sets were combined. The Gold set, the combined Gold–Silver set, and the combined Gold–Silver–Bronze set contained 46, 289, and 2369 sites localized on the decoy amino acid alanine, resulting in recalculated FLR rates of 0.53%, 1.27%, and 6.63%, respectively. These FLR rates represent a satisfactory estimate of false positive rates among the SUMOylated sites identified on lysine in each set.

In addition, we constructed a “non-SUMOylated” set (Supplemental Table S3). First, we selected peptides identified at <1% FDR, then included all lysine residues within these peptides that were not detected as SUMOylated at any FLR threshold. The resulting non-SUMOylated set comprises 65,010 lysine residues on 11,696 proteins.

### Exploring the Structural Properties of SUMOylation Sites

To investigate the structural properties of SUMOylation sites compared to non-SUMOylated lysine residues, we analyzed their tendencies to occur within IDRs and their secondary structural environment. Analysis of IDRs revealed a strong bias of SUMOylated lysines toward disordered regions, with significantly higher disorder proportions observed across all SUMOylated sets compared to the non-SUMOylated set (p-adjusted <0.0001). Specifically, ∼53% of SUMOylated lysines in the Gold set have a per-residue disorder score above 0.5, followed by ∼46% in the Gold–Silver set and ∼43% in the Gold–Silver–Bronze set. In contrast, only ∼30% of lysines in the non-SUMOylated set exceed this threshold (Fig. 1*B*). To further quantify this preference, odds ratios were calculated. In the Gold set, SUMOylated lysines were 2.4 times more likely to be found in disordered regions than in ordered regions, relative to their expected distribution in the human proteome. In contrast, the odds ratio for the non-SUMOylated set is 0.9, suggesting that lysines not modified by SUMO show no strong preference and are nearly evenly distributed between disordered and ordered regions. Because IDRs are typically more solvent-exposed and accessible to modifying enzymes, we hypothesized that SUMOylated lysines would display greater solvent accessibility than non-SUMOylated lysines. Our results show that SUMOylated lysines displayed a significantly higher proportion of exposed sites, with a median increase of ∼14% compared with non-SUMOylated lysines (*p*-value <2.2 × 10^-16^). This increase was strongest for high-confidence (Gold) SUMOylation sites and declined progressively as lower-confidence sites were included (Supplemental Fig. S1). Together, these findings support the idea that the enhanced solvent accessibility characteristic of IDRs provides a putative structural explanation for the observed bias of SUMOylation toward these regions.

Analysis of secondary structure environments revealed significantly lower proportions of SUMOylated lysines within structured regions compared to non-SUMOylated lysines, with ∼15% in the Gold set (p-adjusted <2.4 x 10 − 93), ∼17% in the Gold–Silver set (p-adjusted <7.1 x 10 − 163), and ∼17% in the Gold–Silver–Bronze set (p-adjusted <2.8 x 10 − 187), compared to ∼25% in the non-SUMOylated set. SUMOylation sites are less frequently found in α-helices compared to non-SUMOylated lysines, with the proportion gradually increasing from the Gold to the Gold–Silver–Bronze set, but rising sharply in the non-SUMOylated set, exceeding the expected proportion (dashed blue baseline in Fig. 1*C*). In coiled coils, SUMOylation appears to be markedly diminished, as the proportion remains well below the expected levels (dashed purple baseline) in all SUMOylated sets. This may be due to the tightly packed structure of coiled coils, which could restrict access to SUMOylation machinery, thereby limiting the modification of target lysine residues. In contrast, the proportions of lysines located within turns (dashed red baseline) almost matched the expected background frequency across all sets. Previous studies have reported conflicting findings regarding β-sheets. For example, one study observed an enrichment of SUMOylation in β-sheets compared to background frequency (28), while another reported a significant depletion relative to randomly selected lysine residues from the same proteins (53). In our analysis, the proportions of SUMOylated lysines within β-strands (dashed green baseline) were close to background frequency in the Gold set and showed a slight increase as lower-confidence sites were included in the Gold–Silver–Bronze set. Although the proportion remained higher in the non-SUMOylated set compared with the Gold–Silver–Bronze set, all values remained close to the expected background frequency, with no substantial enrichment or depletion of β-strands across any set.

### Discovery of Associations Between SUMO Sites and Disease-Associated Variants

We integrated SUMOylation site data with human variant annotations from UniProt to assess whether variants occurring at or near SUMOylated lysines are more likely to be disease-associated than those near non-SUMOylated lysines. Given that both the canonical ψKxD/E and inverted canonical D/ExKψ SUMOylation motifs extend up to two residues from the modified lysine, we included all variants mapping to the lysine itself and its two upstream and two downstream flanking positions to ensure coverage of the consensus motif in both orientations.

Our analysis revealed small but statistically higher proportions of disease-associated variants at SUMOylated lysines and their upstream flanking positions compared to non-SUMOylated lysines. The position of the lysine site itself showed significant enrichment in the Gold (5.7%) and Gold–Silver (5.4%) sets, compared to 4.7% in the non-SUMOylated set. The −2 position exhibited significant enrichment across all SUMOylated sets (6.6% in the Gold, 6.3% in the Gold–Silver, and 6.2% in the Gold–Silver–Bronze sets) compared to 5.7% in the non-SUMOylated set. In contrast, the −1 position showed a statistically significant difference but only in the Gold set (6.5%), compared to 5.7% in the non-SUMOylated set (Fig. 1*D*).

Although the absolute differences in variant proportions between the SUMOylated sets and the non-SUMOylated set were small, the consistent enrichment, particularly at the lysine itself and at the −2 position, indicates a pattern that is unlikely to have occurred by chance. All SUMOylation sites mapped to disease-associated variants are provided in Supplemental Table S4. This includes, for instance, a confidently identified SUMOylation site in the Gold set (K647) that overlaps with two *SMARCAL1* variants (c.1939A > C [p.K647Q] and c.1940A > C [p.K647T]), which are associated with severe and mild forms, respectively, of Schimke immuno-osseous dysplasia (SIOD), a rare autosomal recessive disorder, characterized by T cell immunodeficiency, skeletal dysplasia, and renal failure in early childhood (54). In response to replication stress, ATR kinase phosphorylates SMARCAL1 at S652 to downregulate its activity and prevent replication fork collapse (55). This phosphorylation site lies just five residues downstream of K647 and aligns with the PDSM SUMOylation motif (56). K647 is also reported as a ubiquitination site in SMARCAL1, located within its HARP2-SWI/SNF ATPase core domain (57), suggesting that it may serve as a regulatory modification hotspot. The proximity of these PTMs raises the possibility that mutations at K647 disrupt both SUMOylation and ubiquitination, impairing SMARCAL1 function during replication stress and potentially contributing to SIOD pathology.

### Exploration of Potential Crosstalk Between SUMO and Phosphorylation

**Fig. 2:**
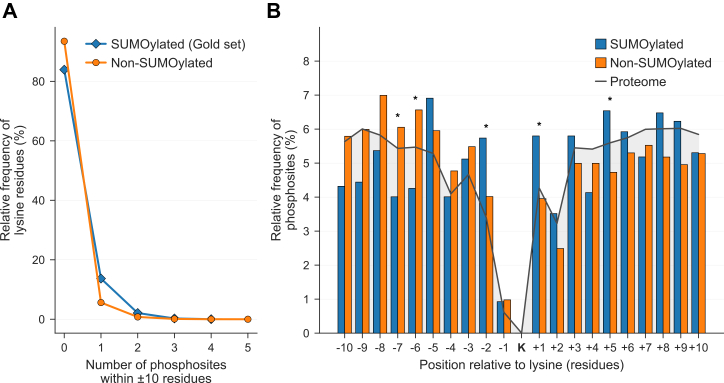
**SUMOylation and phosphorylation co-occurrence**. *A*, distribution of lysine residues by the number of proximal phosphosites within a ±10 amino acid window in the Gold SUMOylated set compared to the non-SUMOylated set. *B*, distribution of phosphosite positions relative to lysine residues within a ±10 amino acid window. The *gray baseline* represents the expected relative frequency of phosphosites in the human reference proteome. Asterisks denote statistically significant differences (∗*p*< 0.05).

We next explored the SUMO atlas data in comparison to other high-quality phosphosite data to understand whether there were clusters of sites co-located, which could be evidence of crosstalk. To investigate whether SUMOylated lysines are more frequently located near phosphorylation sites, we examined the distribution of phosphosites within a ±10 amino acid window of each lysine residue in the Gold SUMOylated set compared to the non-SUMOylated set. We identified 1388 proteins in which SUMOylation and phosphorylation co-occur in proximity. SUMOylated lysines were more likely to have at least one proximal phosphosite than non-SUMOylated lysines. Specifically, 13.9% of SUMOylated lysines in the Gold set had exactly one nearby phosphosite, compared to 5.6% in the non-SUMOylated set. The difference declined sharply for lysines with two proximal phosphosites but remained higher in the SUMOylated set. For lysines with three or more nearby phosphosites, the distributions were nearly identical between the two sets (Fig. 2*A*).

To ensure that this observation was not due to differences in the proteins from which lysines were obtained, we repeated the analysis using a subset of 26,348 non-SUMOylated lysines derived from the same 2742 proteins included in the Gold set. The resulting distribution closely resembled that of the full non-SUMOylated set, supporting the use of the broader protein background in the main comparison (Supplemental Fig. S2).

We evaluated the relative positions of phosphosites around lysine residues to determine whether phosphorylation occurs preferentially at specific positions relative to SUMOylated lysines in the Gold set compared to non-SUMOylated lysines. SUMOylated lysines exhibited higher phosphosite frequencies at positions −2, +1, and +5 compared to non-SUMOylated lysines. The enrichment at position +5 is in agreement with the PDSM motif (ΨKxExxSP) (56). In contrast, non-SUMOylated lysines display higher frequencies at upstream positions −7 and −6 compared to SUMOylated lysines. However, this finding is most likely an artifact of normalization rather than a true enrichment, since all frequencies are relative within each set (SUMOylated, and non-SUMOylated); by having strong enrichment for phosphosites at several positions in the SUMOylated set, by definition other positions (like −6 and −7) show apparent lower frequencies than expected by chance (Fig. 2*B*).

### Discovery of Motifs and Pathways Associated with SUMOylation

We next aimed to explore sequence-based motifs and pathways associated with SUMO sites. First, our analysis of amino acid occurrences at positions −2, −1, +1, and +2 relative to SUMOylation sites identified significant enrichment patterns across multiple datasets. We assessed the Gold, Gold–Silver, and Gold–Silver–Bronze sets, as well as lysine residues not SUMOylated in the non-SUMOylated set.

**Fig. 3:**
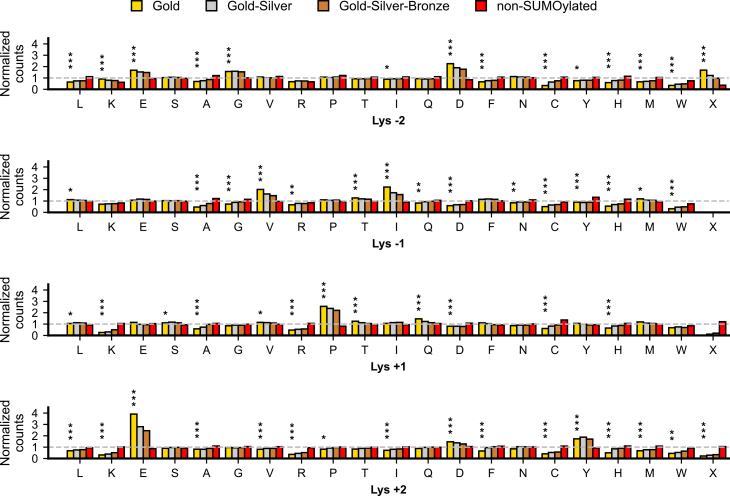
**Normalized amino acid frequencies at proximal positions**. Amino acid frequencies at positions −2, −1, +1, and +2 relative to lysine residues that are SUMOylated in the Gold, Gold–Silver, and Gold–Silver–Bronze sets, or not SUMOylated in the non-SUMOylated set. Reference proteome frequencies for each position are shown by the *dotted gray baseline*. For lysines at the (N-terminus +1), C-terminus, or (C-terminus −1), amino acids at positions −2, (+1 and + 2), or +2, respectively, were labeled as “X” to indicate absence. Asterisks denote statistical significance (∗*p*< 0.05, ∗∗*p*< 0.001, ∗∗∗*p*< 0.0001).

In the Gold set, which represents the most confident SUMOylation sites, aspartic acid (D) displayed a 2.26-fold enrichment at position −2, while isoleucine (I) and valine (V) were enriched at position −1 with fold increases of 2.23 and 2.02, respectively, making them the most enriched amino acids at this position, in agreement with earlier observations (28). At position +1, proline (P) showed a 2.56-fold enrichment, and glutamic acid (E) exhibited the highest enrichment, with a 3.91-fold increase at position +2. These enrichment patterns were statistically significant compared to the non-SUMOylated set, with adjusted *p*-values below 0.0001 (Fig. 3).

The enrichment trends display variability, with some amino acids showing declining enrichment, others increasing, and a few showing inconsistent trends across the Gold, Gold–Silver, and Gold–Silver–Bronze sets. While many amino acids remain consistently higher or lower than the non-SUMOylated set, exceptions exist, highlighting variability in amino acid preferences across different confidence levels of SUMOylation sites. For example, in Figure 3, other amino acids significantly enriched above the 1.5 threshold in the Gold set include glutamic acid (E) and glycine (G) at −2, as well as tyrosine (Y) at +2. While glutamic acid (E) follows the expected declining trend from Gold through to Bronze; the trend is inconsistent for glycine (G) and tyrosine (Y), not following the expected declining trend observed in all amino acids with enrichment higher than 1.5-fold.

In addition, SUMOylated and non-SUMOylated sites in datasets generated using specific protease digestion protocols (Trypsin, LysC/Glu-C, and LysC/Asp-N) were analyzed to test whether different digestion protocols introduced artifactual motifs (Supplemental Fig. S3). The total number of SUMOylated and non-SUMOylated sites varied between the digestion protocols. The Trypsin protocol contained the highest number of sites, with 20,343 SUMOylated and 57,044 non-SUMOylated. In comparison, the LysC/Asp-N protocol included 5370 SUMOylated and 23,779 non-SUMOylated, while the LysC/Glu-C protocol had 1675 SUMOylated and 19,579 non-SUMOylated.

For Trypsin-based datasets, trends for SUMOylated sites are largely aligned with those observed in Figure 3. In contrast, the LysC/Glu-C-based and LysC/Asp-N-based datasets exhibited deviations, likely influenced by their smaller sample sizes. For non-SUMOylated sites, all digestion protocols showed trends closely matching those observed in Figure 3, with minimal deviations. The larger sample sizes for non-SUMOylated datasets likely contributed to this consistency.

Interestingly, SUMOylation appears to be enriched near the N-terminus of proteins. The normalized counts of SUMOylated sites at the N-terminus +1 position (i.e., Lys −2 = X position on Fig. 3) are significantly higher in the Gold set compared to the non-SUMOylated set (p-adjusted ≤0.0006). Unsurprisingly, SUMOylation is not observed at the N-terminus of any protein in the Gold, Gold–Silver, and Gold–Silver–Bronze sets (Lys −1), since the N-terminus of a protein is always methionine in our analysis.

Conversely, SUMOylation shows a significant depletion near the C-terminus (p-adjusted ≤0.0006), with lower normalized counts of SUMOylated lysine residues at the C-terminus and (C-terminus −1) in the Gold set compared to the non-SUMOylated set (i.e., Lys +1 = X and Lys +2 = X positions on Fig. 3, respectively). This depletion may be explained by the requirement for glutamic acid at the +2 position, which is a strong driver of SUMOylation, but cannot be achieved for lysine residues near the C-terminus of a protein. This positional bias might therefore reflect a preference for SUMOylation at lysine residues closer to the N-terminus, while avoiding those near the C-terminus.

In the analysis of disease-associated variants, we observed a strong signal at −2, (as well as in the position of the target lysine), and no statistical significance at +2 position, which is surprising given that the strongest motif enrichment is at +2 position. Given the enrichment of phosphorylation sites at −2, it is possible that the enrichment for disease-associated variants is influenced by some crosstalk between SUMOylation and phosphorylation (at −2).

We identified 31, 60, and 80 SUMOylation motifs in the Gold, Gold–Silver, and Gold–Silver–Bronze sets, respectively (Supplemental Table S5). The Gold set exhibited the highest proportion of motifs with a 3-fold or higher enrichment (18 motifs, 58%), compared to the Gold–Silver set (31 motifs, 52%) and the Gold–Silver–Bronze set (29 motifs, 36%). This higher proportion suggests that the Gold set likely contains truer SUMOylation motifs.

Across all sets, canonical and inverted motifs constitute a significant proportion of the highly enriched motifs. Canonical motifs follow the expected ΨKx[ED] pattern, where Ψ denotes a hydrophobic residue (I, V, L, F, M, A, W, or P), x is any amino acid, and [ED] represents either glutamic acid or aspartic acid. Inverted motifs, meanwhile, follow a reversed orientation, [ED]xKΨ, where [ED] occurs before the lysine residue and a hydrophobic residue follows it. In the Gold set, 13 motifs correspond to the expected canonical ΨKx[ED] motif type, and two align with the inverted canonical [ED]xKΨ motif type; all these motifs exhibit a 3-fold or higher enrichment. The Gold–Silver set contains 12 motifs matching the canonical type, 11 of which have a 3-fold or higher enrichment and 2 inverted motifs (>3-fold enrichment). The Gold–Silver–Bronze set includes 13 canonical motifs (10 with a 3-fold or higher enrichment) and 4 inverted motifs (>3-fold enrichment).

PyMotif-x predicted motifs with a 3-fold or higher enrichment were classified into five types: KxE-type, ExK-type, KxD-type, DxK-type, and unique. In the Gold set, approximately 29% (2485/8639) of SUMOylation sites adhere to the core KxE-type motif. Hendriks *et al*. (2017) found a similar adherence rate of 30% among their top 2000 SUMO sites. When SUMO sites from the Gold–Silver and Gold–Silver–Bronze sets were analyzed, the adherence to the KxE motif dropped to about 21% (4683/22,689) and 18% (6451/35,721), respectively. This trend is consistent with the findings of Hendriks *et al*., who also observed a decline in the core motif adherence as more identified SUMO sites with lower search engine scores were included.

A similar trend was observed for other motif types, although the decline was not as pronounced as for the KxE type. The ExK-type motif adherence decreased from 12.7% (1098/8639) in the Gold set to 11.6% (2642/22,689) in the Gold–Silver set, and 11.3% (4024/35,721) in the Gold–Silver–Bronze set. The KxD-type motif showed a similar pattern, with adherence rates of 6.3% (546/8639), 6.0% (1352/22,689), and 5.5% (1972/35,721) in the Gold, Gold–Silver, and Gold–Silver–Bronze sets, respectively. For the DxK-type motif, adherence dropped from 10.3% (890/8639) in the Gold set to 8.7% (1970/22,689) in the Gold–Silver set, and 8.1% (2893/35,721) in the Gold–Silver–Bronze set.

Motifs that do not fit into any of the predefined groups are classified as unique, several of which share common amino acids such as histidine (H) at either the N-terminus or C-terminus, proline (P), and cysteine (C). In the Gold set, a single highly enriched unique motif, HTGEKPYK, was identified. 294 sites (3.4%) on 224 proteins correspond to the HTGEKPYK motif, all of which belong to the zinc-finger protein family. However, many of the peptides matching this motif mapped ambiguously to multiple homologous zinc-finger proteins due to sequence redundancy, suggesting that the observed motif enrichment may be inflated by peptide-to-protein mapping ambiguity during protein inference rather than being evidence of a truly conserved motif. In the Gold–Silver set, 12 highly enriched unique motifs were observed (HxxEKPxV, HTxEKPxK, HxxEKPxR, HxxxKPxIC, HxxxKPxxC, FSxKxxxxxH, IKP, CxxxxxxKSxxxxH, FxxxxKLxxH, KIxTG, CxKxFK, TKxxxxH). In the Gold–Silver–Bronze set, 10 highly enriched unique motifs were found (HxxEKPxV, HxxEKPxK, HxGxKP, FSxKxxxxxH, FxxxxKLIxH, CxxxxxxKSxxxxH, KIxTG, KHQxxH, KxxGKxF, FxQKxxxxxH).

We next aimed to explore functional roles of SUMO sites, and their potential implication in disease. To this end, we investigated whether disease-associated single amino acid variants occur more frequently at SUMOylated than at non-SUMOylated lysines within specific SUMOylation motifs. Only three motifs, including KxP, the canonical LKxE, and KxD, showed enrichment ratios near or above 1.5-fold, none reached statistical significance after correction for multiple testing (Supplemental Fig. S4), likely due to the limited number of disease variants mapped to individual motifs.

Motifs identified in the Gold set were categorized into parent, child, and orphan motifs based on their hierarchical relationships, as shown in Supplemental Figure S5. Parent motifs (blue) represent broader motifs that encompass multiple specific child motifs (green). Child motifs share a subset of genes with their parents while exhibiting additional sequence variations. In contrast, orphan motifs (gray) exist independently without direct parent-child relationships. To explore these relationships further, we performed Gene Ontology enrichment analysis to investigate whether child motifs acquire functional specialization from their parent motifs and whether orphan motifs are associated with distinct biological processes.

**Fig. 4:**
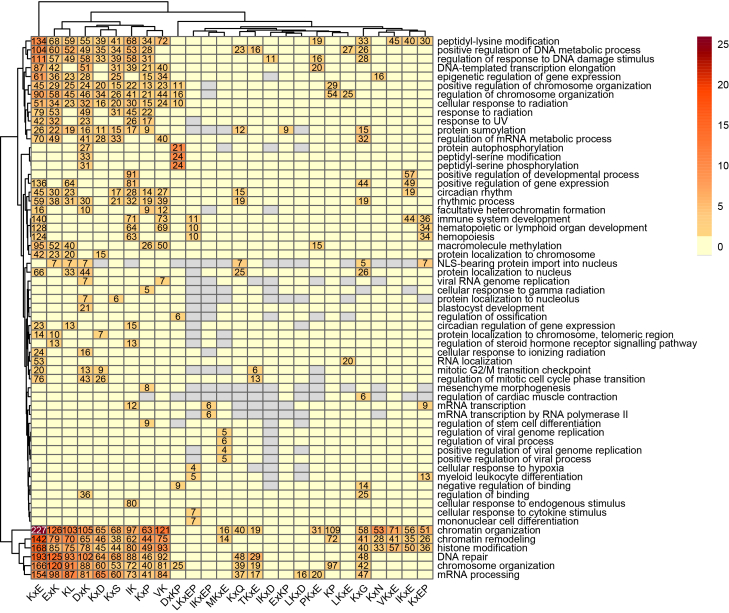
**Heatmap representing the relationship between motifs predicted in the Gold set and their corresponding GO terms**. A gradient from *orange* to *red* reflects the magnitude of significance (−log10(p.adjust)), with *red* indicating the highest significance. Numeric values denote the gene count for significantly enriched terms. *Gray* indicates no genes were mapped to the GO term, and *yellow* indicates insignificant terms (−log10(p.adjust) ≤ −log10(0.05)). Genes encoding proteins associated with the HTGEKPYK, VKxExxE, FKxE, IKQE, and PKxExxE motifs did not yield any significantly enriched terms; hence, these motifs were excluded from the heatmap to maintain simplicity.

GO enrichment analysis revealed significant associations between motifs and key biological processes. GO terms related to chromatin remodeling and organization, peptidyl-lysine and histone modifications, chromosome organization, and mRNA processing were significantly enriched across the majority of motifs, as shown in Figure 4. Among the parent motifs, the IK motif stood out for its exclusive and significant association with the ‘cellular response to endogenous stimulus’ GO term, with 80 mapped genes, indicating a specialized role in internal signaling pathways.

The DxK parent motif and its child DxKP are the only two motifs significantly associated with GO terms related to phosphorylation. Notably, the majority (21 out of 27) of genes mapped to DxK for ‘protein autophosphorylation’ also aligned with the DxKP motif. Similarly, substantial overlaps were observed for ‘peptidyl-serine modification’ and ‘peptidyl-serine phosphorylation,’ with DxK associated with 33 and 31 genes, respectively, and DxKP with 24 genes for both terms. Beyond phosphorylation, DxKP was exclusively and significantly associated with the ‘regulation of ossification’ GO term, suggesting a specialized role in bone formation processes.

The KxE parent motif primarily shared GO terms with other motifs; however, many of its child motifs exhibited distinct functional roles. For instance, the KxEP child motif was exclusively and significantly associated with the ‘myeloid leukocyte differentiation’ GO term. Similarly, the MKxE child motif was uniquely and significantly linked to GO terms related to the regulation of viral processes. Additionally, the LKxEP child motif showed exclusive and significant associations with GO terms including ‘cellular response to cytokine stimulus,’ ‘mononuclear cell differentiation,’ and ‘cellular response to hypoxia.’ These findings demonstrate the functional specialization of child motifs within the broader context of the parent motif.

Among the orphan motifs, only KxP and KxG exhibited distinct associations with specific biological processes. The KxP motif was exclusively and significantly linked to several GO terms, including ‘regulation of stem cell differentiation,’ ‘mesenchyme morphogenesis,’ and ‘cellular response to gamma radiation.’ Similarly, the KxG motif was uniquely and significantly associated with the ‘regulation of cardiac muscle contraction.’ These findings highlight the unique contributions of these two orphan motifs to specific biological processes.

Overall, the results provide new insights into the hierarchical and independent roles of motifs. While some motifs acquire functional specialization, the majority contribute to broader, shared cellular processes, particularly those occurring in the nucleus.

### SUMOylation and Ubiquitination Comparative Analysis

**Fig. 5:**
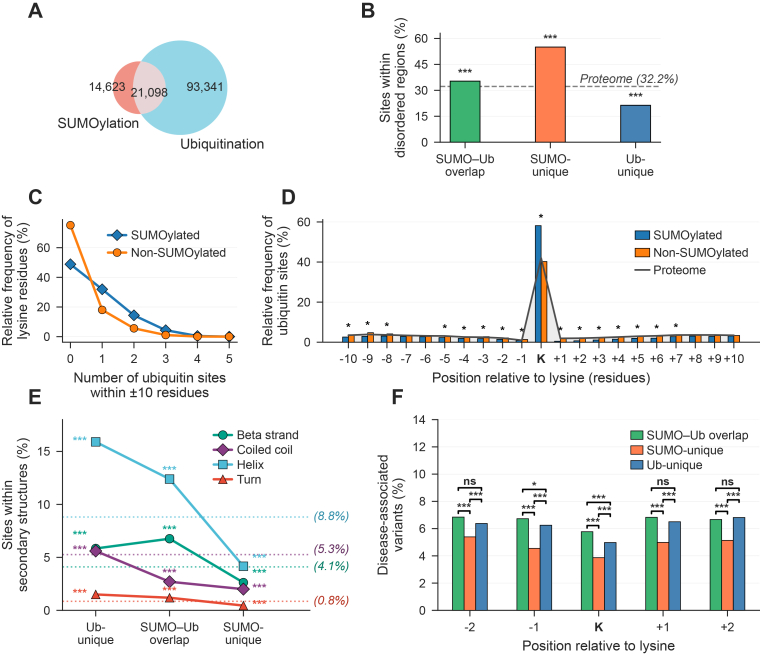
**SUMOylation and ubiquitination site overlap and associated structural and functional features**. *A*, Venn diagram showing the overlap between SUMOylated and ubiquitinated lysine sites. *B*, Proportions of lysine residues with a per-residue disorder score >0.5 across SUMO–Ub overlap, SUMO-unique, and Ub-unique site categories. The dashed baseline represents the expected proportion of disorder in the human reference proteome. *C*, distribution of lysine residues by the number of proximal ubiquitin sites within a ±10-residue window for Gold SUMOylated compared to non-SUMOylated lysines. *D*, Positional distribution of ubiquitin sites relative to the central lysine within a ±10-residue window. The *gray* baseline represents the expected frequency in the human reference proteome. *E*, Proportions of sites within each secondary structural element (β-strand, coiled coil, α-helix, and turn). *Dashed lines* indicate proteome background proportion for each element. *F*, Proportions of disease-associated variants at the lysine position and at flanking positions (−2, −1, +1, +2). Brackets indicate pairwise chi-squared test comparisons. Asterisks denote statistical significance (∗∗∗*p*< 0.001, ∗*p*< 0.05).

Given that both SUMOylation and ubiquitination target lysine residues, we next explored potential crosstalk or competition between SUMO sites identified in this work, with a recent large-scale ubiquitination build created using similar methods. Both builds apply the same Gold–Silver–Bronze tiering system (24), where Gold sites are observed in two or more datasets with an FLR below 1%, Silver in one dataset with an FLR below 1%, and Bronze sites do not meet the Gold or Silver criteria but have an FLR below 5%. Of the lysine residues identified across both builds, 21,098 sites were modified by both SUMO and ubiquitin (SUMO–Ub overlap), while 14,623 were exclusively SUMOylated (SUMO-unique) and 93,341 were exclusively ubiquitinated (Ub-unique) (Fig. 5*A*). Sites classified as SUMO-unique, Ub-unique or SUMO–Ub overlap are available in Supplemental Table S6.

We identified 30, 36, and 48 SUMOylation motifs in the SUMO-unique, SUMO–Ub overlap, and Ub-unique sets, respectively (Supplemental Fig. S6). SUMO-unique motifs showed considerably higher enrichment than both Ub-unique and SUMO–Ub overlap motifs. The most highly enriched SUMO-unique motifs included HxxxKPxVC (∼31-fold), IKxEP (∼21-fold), CxKxxxxKxxxxxH (∼18-fold), HxxxKPxxC (∼12-fold), LxKHxxxH (∼11-fold), VKxE (∼9-fold), and IKxE (∼9-fold). SUMO–Ub overlap motifs exhibited modest enrichment (∼1.3–4-fold), except for one motif (HTGEKPYK, ∼14-fold). In contrast, Ub-unique motifs showed weak enrichment (∼1.1–1.6-fold), consistent with the absence of a clear consensus motif for ubiquitin (58). When compared with SUMOylation motifs derived from the full SUMO build (Gold-Silver-Bronze), the SUMO–Ub overlap set shared 26 motifs with the full SUMO build, whereas the SUMO-unique set shared only 15 motifs. However, for shared motifs found in both sets, the SUMO-unique set showed stronger fold enrichment, particularly for canonical and inverted SUMO consensus motifs (VKxE, IKxE, DxKP, PKxE, ExKP, FKxE).

We analyzed the proportions of SUMOylation and ubiquitination sites located within IDRs (Fig. 5*B*). SUMO-unique lysines showed the highest proportion of sites located within IDRs (∼55%), significantly exceeding the proteome background (32.2%; adjusted *p*< 1 × 10^-16^). This proportion is consistent with that observed in the full SUMO build (∼53%). SUMO–Ub overlap sites exhibited 35% of lysines within IDRs, slightly above the proteome background (adjusted *p*= 6.48 × 10^-21^). In contrast, Ub-unique lysines were significantly depleted in IDRs, with ∼21% of sites located in disordered regions (adjusted *p*< 1 × 10^-16^).

We identified 1785 proteins in which SUMOylation and ubiquitination co-occur in proximity. SUMOylated lysines were more likely to have at least one proximal ubiquitination site (51.2%) than non-SUMOylated lysines (28.8%) (Fig. 5*C*). We next evaluated the relative positions of ubiquitination sites around lysine residues to determine whether ubiquitination occurs preferentially at specific positions relative to SUMOylated lysines (Fig. 5*D*). The highest ubiquitination frequency was observed at position 0 (the lysine residue itself) for SUMOylated lysines (58.1%) compared with non-SUMOylated lysines (40.2%). Although several relative positions showed statistically significant differences, the magnitude of enrichment was small compared with that observed at position 0. These results indicate that co-occurrence of SUMOylation and ubiquitination most frequently involves modification of the same lysine residue rather than adjacent positions.

We analyzed the proportions of lysine sites within secondary structural elements (Fig. 5*E*). The Ub-unique set showed the highest proportions of sites within helices (15.9%), coiled coils (5.6%), and turns (1.5%), while SUMO–Ub overlap sites exhibited the highest proportion within β-strands (6.8%). In contrast, the SUMO-unique set displayed reduced proportions across all four structural elements (helices: 4.2%; β-strands: 2.6%; coiled coils: 2.0%; turns: 0.5%) relative to the proteome baseline. These proportions were also lower than those observed in the full SUMO build (helices: 9.0%; β-strands: 5.1%; coiled coils: 2.4%; turns: 0.9%). All differences from the corresponding proteome background proportions were statistically significant (all adjusted *p*< 0.001).

To assess whether variants occurring at or near lysines are more likely to be disease-associated, we compared the proportion of disease-associated variants at lysine residues and their ±2 flanking positions across the three site categories (Fig. 5*F*). The SUMO-unique set showed the lowest proportion of disease-associated variants at all five positions, significantly below both the SUMO–Ub overlap and Ub-unique sets (all pairwise adjusted *p*< 0.001). The SUMO–Ub overlap set showed significantly higher proportions of disease-associated variants than the Ub-unique set at the lysine residue itself (adjusted *p*= 2.5 × 10^-5^) and at the −1 position (adjusted *p*= 0.047). We can thus conclude that while SUMO and ubiquitin generally target different regions of proteins (based on disorder and secondary structure analysis), where they do target the same sites, these are enriched in functionally significant sites, as supported by the disease-associated variant analysis.

## Discussion

Our study provides a comprehensive reanalysis of existing human SUMOylation datasets to generate a high-confidence resource for SUMOylated lysine sites. By independently estimating the global FLR using a decoy residue strategy, we generated comparable site-level confidence assessments across datasets. We further classified merged SUMOylated sites identified at <5% FLR within each dataset into three confidence sets (Gold, Silver, and Bronze) to reduce the risk of over-interpreting lower-confidence identification findings in downstream analyses.

A key challenge in the reanalysis of SUMOylation site data is the absence of a standard experimental protocol. Our approach addresses this by accounting for differences in SUMO mass remnants (footprints) and the specific proteolytic enzymes used across datasets during database searching. Variability in enrichment strategies and digestion protocols can introduce biases, which we aimed to mitigate through consistent processing and filtering.

In many previously published studies, particularly those focusing on training SUMOylation site prediction models, the non-SUMOylated negative set is typically constructed by treating any lysine residue that is not annotated as SUMOylated in databases or published literature as non-SUMOylated, without verifying whether the residue was ever experimentally observed in MS-based analyses (59, 60, 61). The non-SUMOylated set in this study was extracted from the same peptide pool used to identify SUMOylated sites. To minimize the inclusion of ambiguous residues, only lysine residues observed in the reanalyzed MS experiments on peptides detected below 1% FDR and not identified as SUMOylated at any FLR threshold across all datasets were included.

We performed a comparative analysis of SUMOylated and non-SUMOylated sites to better understand the sequence, structural, and functional features that make a lysine “SUMOylatable.” By isolating distinguishing properties with biological relevance, we aimed to provide an AI-ready reference set of SUMOylation and non-SUMOylation sites. Example uses of the data could include training and benchmarking of improved SUMOylation site prediction models, improving scoring of SUMO identification from MS data, or for example developing a score for determining SUMO sites with the strongest functional consequences, similar to work performed for phosphorylation (62). Data sets showing the overlap and potential competition between SUMOylation and ubiquitin will also be useful for these and related purposes, such as defining PTM hot spots.

Finally, the availability of the human SUMO build through established proteomics platforms, including UniProtKB, PeptideAtlas, and PRIDE, encourages further experimental validation, computational analysis, and machine learning-based prediction of SUMOylation sites.

## Data Availability

**Fig. 6:**
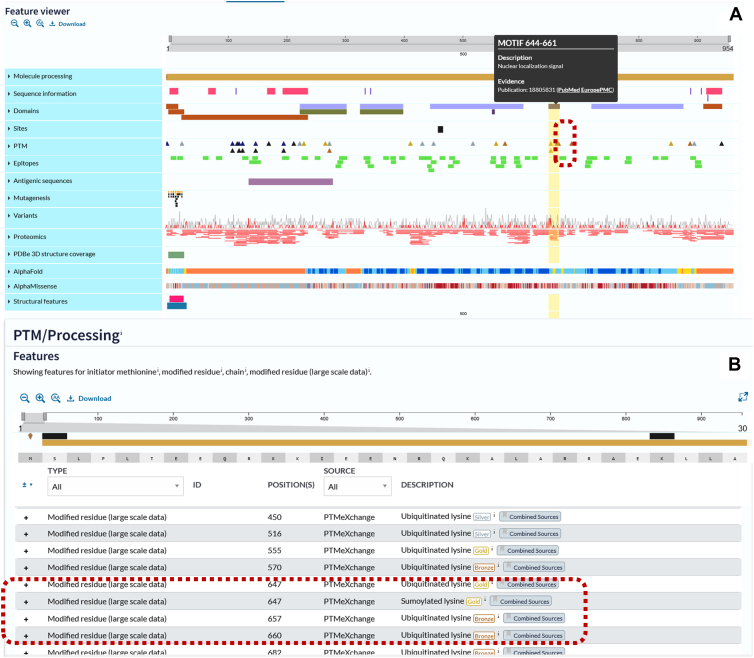
**The SUMOylation build is available in UniProtKB for exploration and integration with other stored data types**. *A*, screenshot from UniProtKB *Feature Viewer* (www.uniprot.org/uniprotkb/Q9NZC9/feature-viewer) showing PTMs and other data types as tracks for SMARCAL1 (Q9NZC9). *B*, screenshot from the UniProtKB *Entry view* (https://www.uniprot.org/uniprotkb/Q9NZC9/entry#ptm_processing) showing the table of SUMOylation sites and other PTMs. Both panels highlight SUMOylation and ubiquitination sites, including those mapped to disease-associated variants, overlapping a nuclear localization signal. UniProtKB © UniProt Consortium (CC BY 4.0).

The Human SUMO build is one of several PTM-specific builds generated as part of the PTMeXchange project (https://www.proteomexchange.org/ptmexchange/index.html). It supports high-quality reanalysis of MS/MS datasets enriched for specific PTMs and facilitates open access to the reanalyzed data. The MS proteomics data have been deposited to the ProteomeXchange Consortium via the PRIDE (63) partner repository with the dataset identifier PXD068489. The data have also been integrated into UniProtKB (Fig. 6), allowing SUMOylation site evidence to be viewed as part of the protein feature viewer, and are also downloadable from the UniProt Proteins API.

Additionally, the SUMO build has been released via PeptideAtlas (https://peptideatlas.org/builds/human/sumo/), enabling more detailed examination of evidence at the level of individual proteins, peptidoforms, and spectra (Supplemental Fig. S7). Each site in our data is linked to its supporting spectrum via a Universal Spectrum Identifier (USI) (64), which can be queried at ProteomeCentral (https://proteomecentral.proteomexchange.org/usi/) to access spectral evidence across multiple repositories in order to evaluate potential alternative interpretations, including other peptide matches or modification localizations.

All analysis scripts and example input files are openly available at https://github.com/PGB-LIV/Human-SUMO-Analysis. The repository includes Python scripts for generating custom peptide databases, an R script to remove diagnostic SUMO remnant fragment ions from mzML files, Python scripts for post-mzidFLR processing and for classifying SUMO sites into quality tiers, Python scripts to extract 15-mer sequences for motif analysis, and scripts to generate figures presented in the manuscript.

## Supplemental Data

This article contains supplemental data.

## Conflict of Interest

The authors declare no competing interests.
