# Supplementary material for: A Landscape Analysis of Human SUMOylation
Source: Mol Cell Proteomics. 2026 Apr 20;25(5):101571. doi: 10.1016/j.mcpro.2026.101571 (PMC13213312; doi:10.1016/j.mcpro.2026.101571)
Supplement: Supplemental Material 7 [file mmc7.pdf]

Supplementary Figures

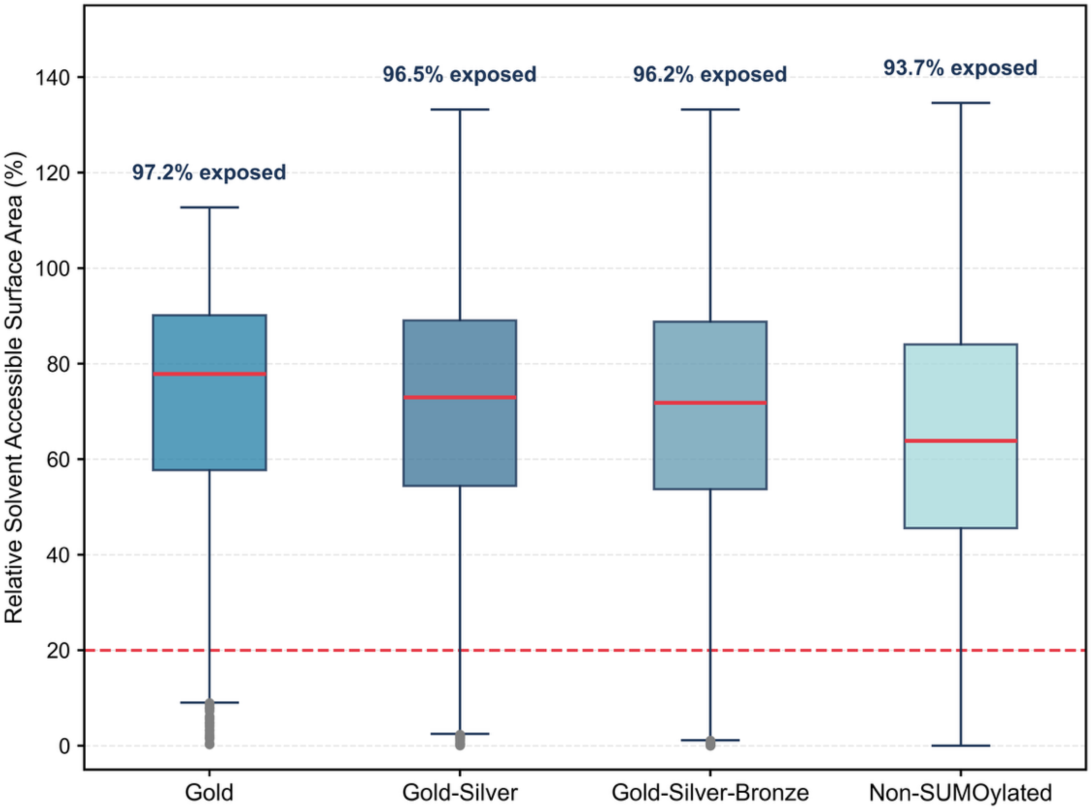

Fig. S1. Distribution of Relative Solvent Accessibility (rASA) values for lysine residues in SUMOylated (Gold, Gold-Silver and Gold-Silver-Bronze) and Non-SUMOylated sets. The red dashed line represents the 20% rASA threshold used to classify residues as buried or exposed. Percentages above each boxplot indicate the proportion of exposed lysine (rASA ≥20%).

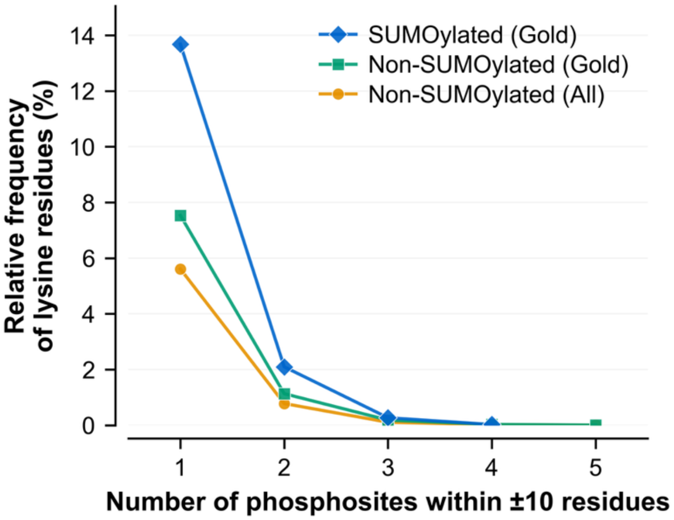

Fig. S2. Distribution of lysine residues by number of proximal phosphosites within a ±10 amino acid window. Lysines in the Gold SUMOylated set were compared to two non-SUMOylated lysine sets: (1) the full non-SUMOylated set, comprising 65,010 lysine residues from 6,992 proteins, constructed by selecting peptides identified at <1% FDR and including all lysines not detected as SUMOylated at any FLR threshold; and (2) a protein-matched subset of 26,348 non-SUMOylated lysines detected at any FLR across the same 2,742 proteins included in the Gold set.

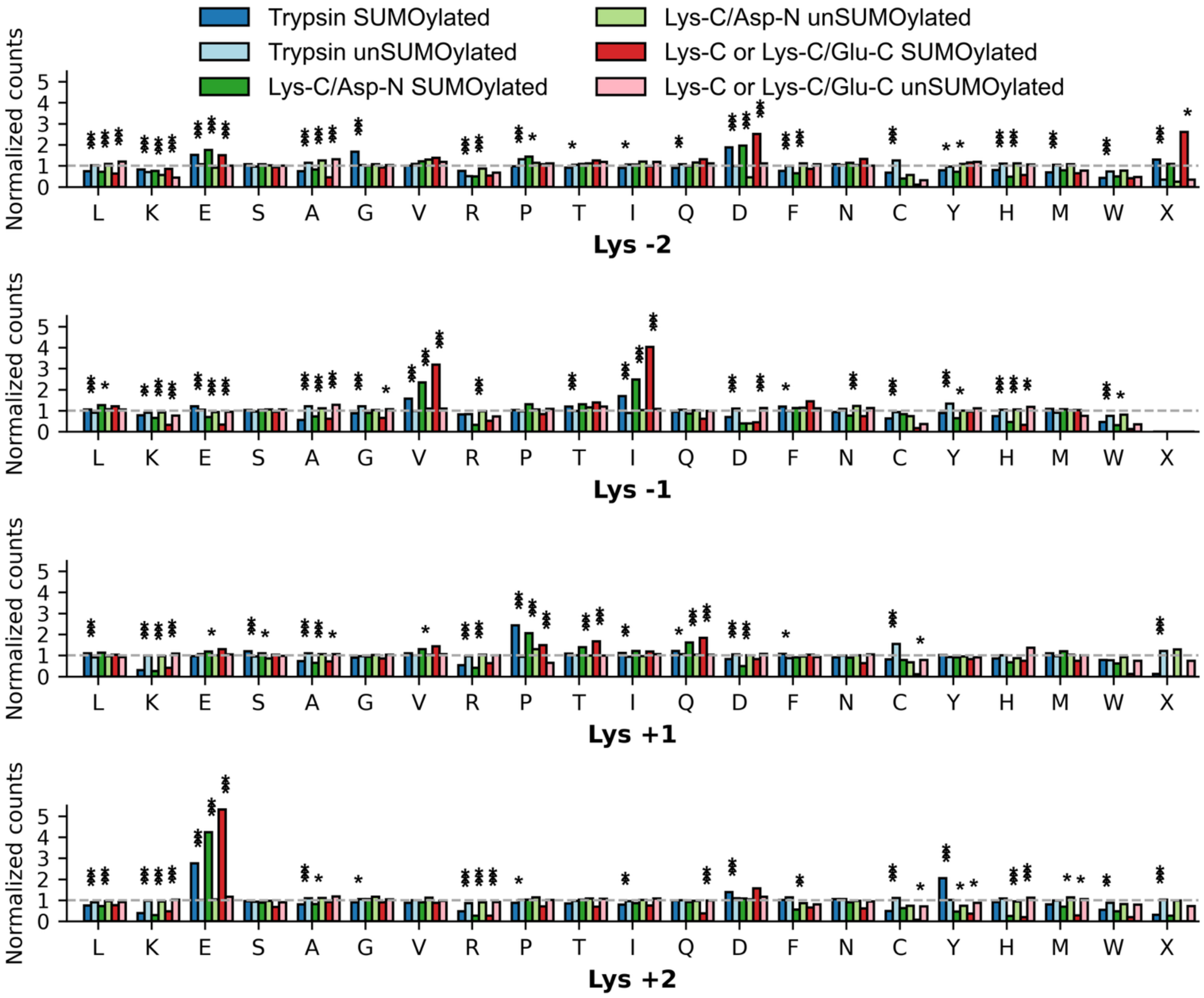

Fig. S3. Normalized amino acid frequencies at proximal positions, grouped by digestion protocol. SUMOylated sites detected below 1% FLR, divided into three sets based on the digestion protocol used, and lysine residues not SUMOylated at any FLR in their corresponding non-SUMOylated sets. Reference proteome frequencies for each position are shown by the dotted gray baseline. For lysines at the (N-terminus +1), C-terminus, or (C-terminus −1), amino acids at positions −2, (+1 and +2), or +2, respectively, were labeled as “X” to indicate absence. Asterisks denote statistical significance (*$p < 0.05$, **$p < 0.001$, ***$p < 0.0001$).

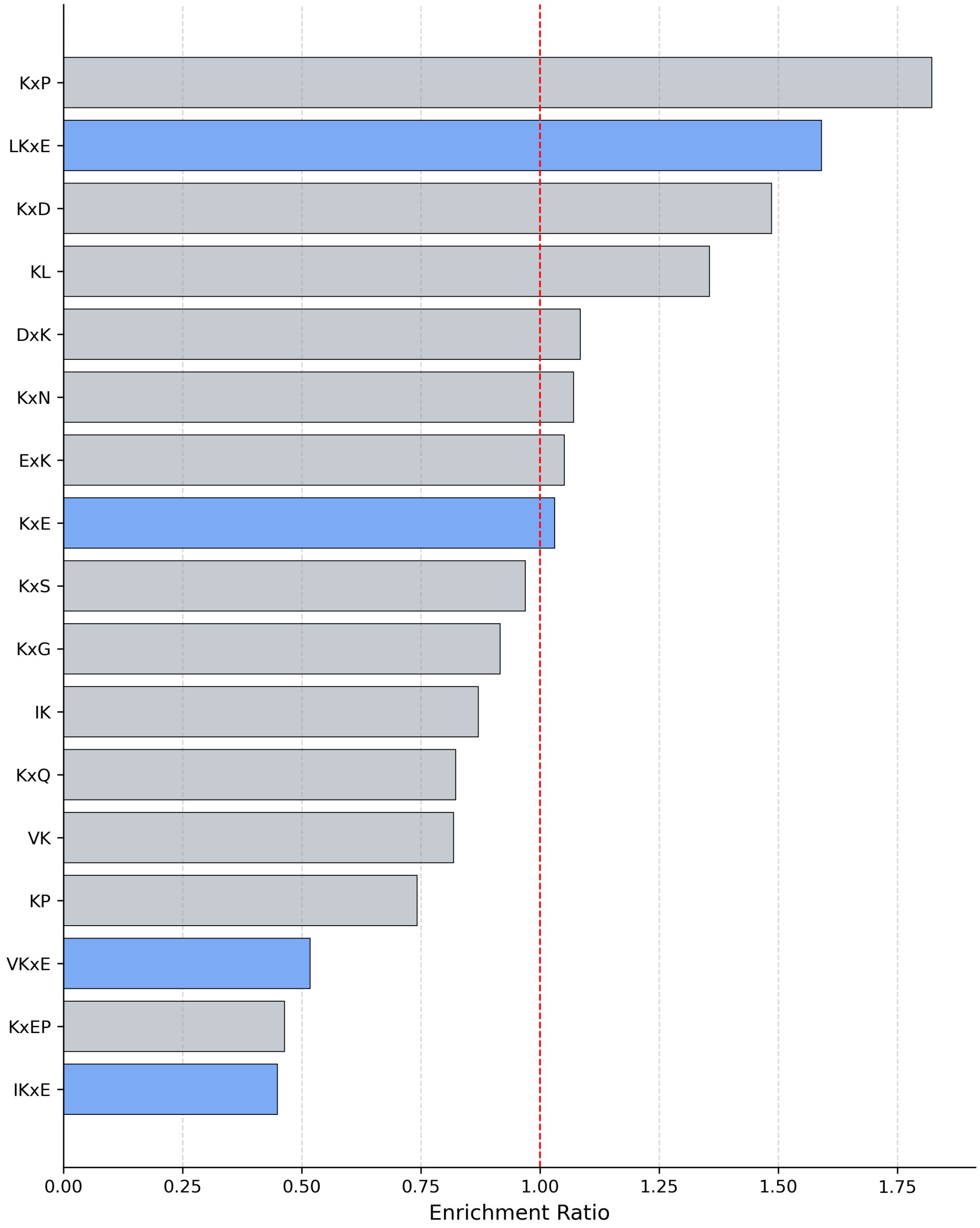

Fig. S4. Enrichment of disease-associated lysine variants across different SUMOylation motifs. The enrichment ratio of disease-associated lysine variants at SUMOylated sites compared to non-SUMOylated lysine for each identified SUMOylation motif. The canonical SUMOylation consensus motifs (ΨKxE) and the minimal core KxE are highlighted in blue. Only motifs containing >200 SUMOylation sites are shown; HTGEKPYK was excluded due to zero disease variants mapped. An enrichment ratio of 1 is represented by the red dashed line.

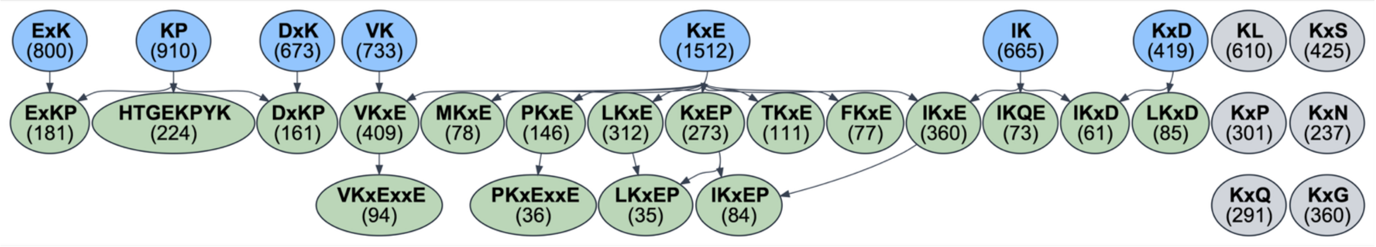

Fig. S5. Hierarchy of the 31 motifs identified in the Gold set. Seven core motifs (parent, blue) form the foundation of the hierarchy, with 18 motifs (child, green) nested underneath. The remaining six motifs (orphan, gray) exist independently, without a parent or child relationship. The counts of motif-containing proteins are shown in parentheses next to each motif.

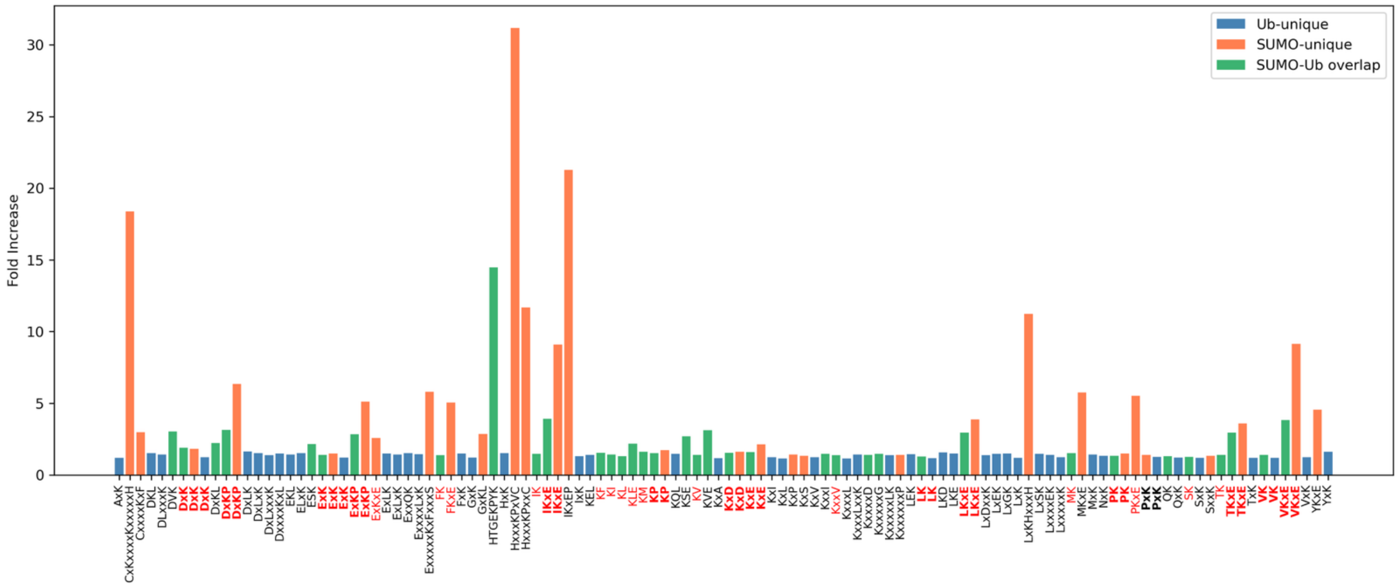

Fig. S6. Fold enrichment of sequence motifs identified in the SUMO-unique, SUMO–Ub overlap, and Ub-unique sets. Motifs present in more than one set are shown in bold, and those also predicted using the full SUMO build (Gold–Silver–Bronze sites) are highlighted in red. Only motifs appearing in at least 0.5% of the total sites within each set were retained for comparison.

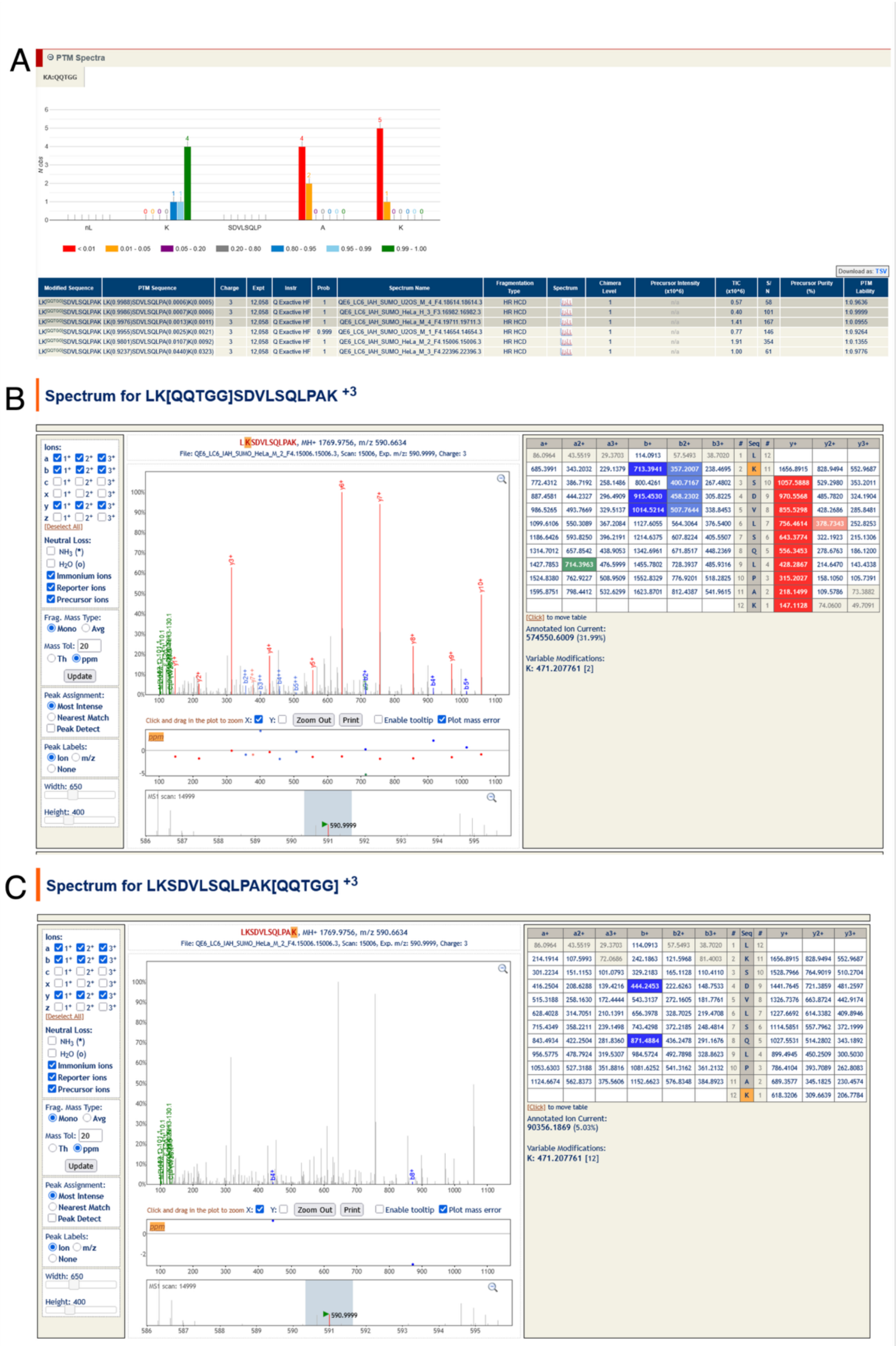

Fig. S7. The SUMOylation build is available in PeptideAtlas for exploration of evidence down to the level of all individual spectra, linked via the Universal Spectrum Identifier (USI) framework. A, Multiple spectra supporting SUMOylation of lysine at position 647 of SMARCAL1 (Q9NZC9), while none support SUMOylation of lysine at position 657. **B**, Example spectrum demonstrating strong fragment ion matches supporting correct localization of SUMOylation on K647 (USI: mzspec:PXD004927:QE6_LC6_IAH_SUMO_HeLa_M_2_F4:scan:15006:LKSDVLSQLPA[QQTGG]K/3). **C**, No evidence for SUMOylation at K657: mzspec:PXD004927:QE6_LC6_IAH_SUMO_HeLa_M_2_F4:scan:15006:LKSDVLSQLPAK[QQTGG]/3).
